# Supplementary material for: The dolutegravir failure cohort: A multi-country longitudinal cohort with a randomised clinical trial of continued dolutegravir versus switch to darunavir in people with viraemia while on dolutegravir in Sub-Saharan Africa (The Ndovu Study) protocol
Source: PLoS One. 2026 Mar 13;21(3):e0330792. doi: 10.1371/journal.pone.0330792 (PMC12987441; doi:10.1371/journal.pone.0330792)
Supplement: S4 File — (DOCX) [file pone.0330792.s004.docx]

**Clinical Protocol**

**Investigating the optimal management of dolutegravir resistance: a multi-country cohort study**

**Short Title: Ndovu Cohort Study**

**ClinicalTrials.gov Identifier: NCT06762054**

**Sponsor: University of Nairobi**

This is a multi-country study led by the University of Nairobi, acting as Sponsor, and collaborating with Instituto Nacional de Saúde (INS) Mozambique, Muhimbili University of Health and Allied Sciences (MUHAS) Tanzania, SolidarMed Lesotho, and the London School of Hygiene and Tropical Medicine United Kingdom

**Funding: Bill & Melinda Gates Foundation**

**Protocol Version 1.2: 24-February-2025**

**Investigators**

**Chief Investigator**

Loice Achieng Ombajo, MBChB, M.Med, DLSHTM, MSc (ID), FRCP

**Principal Investigators**

Principal Investigator, Mozambique: Nalia Ismael, BSc, MSc, PhD

Principal Investigator, Tanzania: Patricia Munseri, MD, MMed, MPH, PhD

Principal Investigator, Lesotho: Irene Ayakaka, MBChB, MPH, MRes

**Co-Investigators**

Co-investigators – Kenya:

Jeremy Penner, MD, MHSc, DTM&H, CCFP, FCFP

Emily Wangui Kamau, MBChB, M.Med, MSc (ID)

Patrick Amoth, MBChB, M.Med

Andrew Mulwa, MBChB, Msc

Elizabeth Abong’o, KRCHN

Leonard Kingwara, BSc, MPH, PhD

Dalton C. Wamalwa, MBChB, M.Med, MPH

James Wagude, MBChB, M.Med

Rose Wafula, MBChB, MPH

Lazarus Momanyi, MBChB, MPH

Joseph Nkuranga, MBChB, MSc (Epi)

Florentius Ndinya, MBChB, M.Med,

Anne-Marie Macharia, MBChB, M.Med

Simon Wahome, BPharm, MPharm

Anthony Kiplagat, DCM, BA, MSc

Caroline Wafula, BPharm, MPharm, MBA

Lisa Abuogi, BA, MD, Res, MSc

Rena Patel, BA, M Phil, MD, MPH

Co-Investigators – Mozambique:

Raquel Matavele Chissumba, BSc, MSc, PhD

Patricia Maria Ramgi, MD

Co-Investigators – Tanzania:

Muhammad Bakari, MD, M.Med, PhD

Jamila Said Didi, MD, MBA, M.Med, MSc (Nephrology)

Co-Investigators – Lesotho:

Niklaus Labhardt, MD, MIH, FMH

Anna Klicpera, MD, DTM, MSc

Tapiwa Tarumbiswa MBBS, MBA

Co-Investigators – London School of Hygiene and Tropical Medicine (LSHTM):

Daniel James Grint, BSc, MSc, PhD

Charles Opondo, BPharm, MSc, PhD

**Protocol Amendment Summary of Changes Table**

**Document History**

| Protocol Version | Date |
| --- | --- |
| 1.0 (Original version) | 04-October-2024 |
| 1.1 | 27-November-2024 |
| 1.2 | 24-February-2025 |

**Amendment 1: Protocol Version 1.1, 27-November-2024**

The changes made to version 1.0 of the protocol are summarized below:

| **Section and Page Number** | **Description of Change** | **Brief Rationale** |
| --- | --- | --- |
| Cover page and footers | Updated protocol version and date | To differentiate the versions of the protocols |
| Abstract, Page iv | Updated to include the target sample size, include enhanced adherence counseling and describe analysis methods | Enrich the abstract in-line with review comments |
| Figure 1 Ndovu Cohort Study Schema, Page 8 and Appendix 2 | Edits made to the schema to align with other sections of the protocol | To align with other sections of the protocol |
| Table 3: Time and Events Table, Page 18 | Included measurement of head circumference and descriptions of who will have height/length, MUAC and head circumference measured | Updated to better describe the anthropometric measurements that the participants will undergo during the study |
| Assessment at Each Visit, Pages 19 and 21 | Added description of how head circumference will be measured and the protocol definition of girls or women of child-bearing potential | Standardization of how head circumference will be measured and to clearly define WOCBP who will have pregnancy testing |
| Ndovu Cohort Site Specific Addendum for Kenya | - Included additional sites as updated in appendix 1  - Included section on data privacy as per Kenyan Law | - To align with protocol  - In response to ERC review comments |
| Participating Study Sites, Appendix 1 | Updated sites list for Kenya as detailed below:  - Included additional satellite health facilities: St Mary’s Mission Hospital, KU Teaching and Referral Hospital as one of the sites in Kiambu, Tudor Sub-County, Kayole 1 Health Center, Port-Reitz Sub-County and Likoni Sub-County Hospital  - Edited Kayole 2 Health Center to read Sub-County Hospital | Additional facilities were included to maximize the number of potential participants from high prevalence counties; correction of the identity of one of the facilities |
| Participant Information Sheets and Consent forms, Appendices 4A,4B,4C | Labelled each information sheet and consent form including ages in which each should be used  Included additional measurements to be taken as per protocol: head circumference  Edited grammar  Edited quantity of samples to be drawn from 30mls to 48mls. | To minimize incorrect application of information sheets; to update study measurements as per protocol; for clarity and facilitating better understanding; and to align with protocol |
| Case Report Forms, Appendix 5 | Edits and corrections made as detailed below:  - Included additional health facilities in site codes  - Included head circumference as an anthropometric measurement  - Added HIV history on DTG DRMs and an entry route for eligibility screening for RCT if these are present  - Included ABC,3TC, NVP and AZT/3TC/LPV/r as ARV regimens to be selected from a drop-down list  - Added dose and frequency of dose to regimen selected for prescription  - Removed delaying enrollment into study because of TB diagnosis  - Included whether pregnancy was done and results of pregnancy test into follow up CRF  - Included clinical/medical causes of impaired adherence-poor absorption, inadequate dosing (paediatrics), included options for barriers assessed and plans to mitigate them in individualized treatment plan check list and additional barriers  - After number 7 in italic corrected to VL ≥ 200 copies/mL (instead of 400**)**  **-** NRTI resistance: include “Resistance to abacavir, Zidovudine and 14: for first “I” included as it was missing in "INSTI" | Edits made to align with the protocol and also made corrections identified during review of the CRFs |

**Ammendment 2: Protocol version 1.2, 24^th^ February 2025**

| **Section and Page Number** | **Description of Change** | **Brief Rationale** |
| --- | --- | --- |
| List of co-investigators, page 2 | Updated list of co-investigators to include Dr Patrick Amoth, Dr Andrew Mulwa, and Elizabeth Abong’o | Added co-investigators to address Ministry Leadership and Policy implications of the study |
| Abbreviations and Acronyms, page 9 | AZT-Azidothymidine or Zidovudine | Corrected a typographical error |
| Section 2.2 Secondary Objectives | Added two secondary objectives: To assess viral suppression based on prior PI or INSTI exposure and To assess viral suppression based on prior PI or INSTI failure | To align with changes in the inclusion criteria that include participants with suspected or confried treatment failure. |
| Section 3, Figure 1, Page 8 | Updated the cohort schema in-line with the change in the exclusion criteria in section 4.5.1 | To align with modification in exclusion criteria |
| Section 4.5.1  Exclusion Criteria | p.10 modified exclusion criteria to include participants who had suspected or confirmed treatment failure while on PIs or INSTIs by deleting requirement that excluded participants with suspected or confirmed treatment failure while on PIs or INSTIs | To include participants on second-line or third-line regimens who are on dolutegravir into the Cohort Study |
| Section 4.5.2 Country-Specific Site Description and Recruitment Strategy Table 2 Country Specific Site Description p.14 | p.14 deleted section that required exclusion of participants with prior suspected or confirmed treatment failure while on PIs or INSTIs in the pre-screening strategy circumference will be measured and the protocol definition of girls or women of child-bearing potential | To align with modification in exclusion criteria |
| Section 7.2 Informed Consent, Page 25 | Included a request for waiver of parental consent for adolescents aged 15 to 17 years old who are not accompanied by their caregivers | In many settings, adolescents are unaccompanied by their caregivers whereas this study is minimal risk as it does not deviate from the standard care |

**Abstract**

**Background**

The majority of people living with HIV (PLWH) on first line antiretroviral therapy (ART) in low and middle-income countries are on dolutegravir (DTG)-containing regimens. Different countries have adopted different approaches in the management of people on DTG-based first line ART with repeat HIV viral load (VL) of > 1,000 copies/mL after 3 months of enhanced adherence counseling. For example, Kenya recommends a drug resistance test (DRT) to guide on switch and the optimal second line regimen; Mozambique and Tanzania recommend switch to 2 nucleoside reverse transcriptase inhibitors (NRTIs) and protease inhibitors (PIs) without drug resistance testing; South Africa does not recommend switch from DTG or DRT for those who are on first-line DTG-containing regimens within the first 2 years of treatment, after which management is guided by possible DRT and expert opinion. The World Health Organization has recognised the role of drug resistance testing (DRT) in a treatment failure algorithm for people living with HIV receiving DTG-based treatment to minimise unnecessary switches from this regimen. The switch to PI has disadvantages including higher cost, higher pill burden, less convenient administration (often should be taken with food), more potential drug-drug interactions, poorer tolerability and more long-term toxicities.

**Objectives**

To assess viral suppression rate following enhanced adherence counseling among people on DTG-based ART who have sustained viraemia (≥ 1,000 copies/mL) after at least six months on ART.

**Methods**

This is a multi-country observational prospective cohort study over 12 months describing HIV-1 viral suppression in people with high viral load (≥ 1,000 copies/mL) after at least six months on DTG-based ART. The Study targets to enrol 6,600 participants in Kenya, Mozambique, Tanzania and Lesotho. Study visits and VL testing will take place at enrolment and then every 3 months for up to 12 months during the active follow-up period for participants who do not achieve viral suppression < 200 copies/mL. During each visit, protocol-specified enhanced adherence counseling and assessment/management of other causes of viremia will continue for at least 3 sessions. For participants who achieve the primary outcome of HIV-1 RNA < 200 copies/mL during the active follow-up period, a repeat VL will be performed after 3 months; outcomes from routinely collected program data (viral load, loss to follow-up, death) will be collected 12-24 months from enrolment to assess durability of suppression among this group. We will estimate the viral suppression at 6 and 12 months using a generalized linear regression model with binomial distribution as well as assess for predictors of achieving suppression, development of DTG-associated drug resistance mutations (DRMs), and development of opportunistic infections using logistic regression models. Participants will also be assessed for eligibility to enrol into a nested randomized clinical trial (RCT) on management of people who develop DRMs during the cohort study (Ndovu RCT; see separate protocol).

**Study Utility**

This study will address the gap in published data on viral suppression among people meeting criteria for virologic failure on DTG-based ART regimens without a change in regimen, and prospective data on emergence of DTG-associated DRMs and the impact of those DRMs on suppression.

**Abbreviations/Acronyms**

| 3TC | Lamivudine |
| --- | --- |
| ABC | Abacavir |
| AIDS | Acquired Immune Deficiency Syndrome |
| ART | Antiretroviral therapy |
| ATV/r | Ritonavir-boosted atazanavir |
| AZT | Azidothymidine or Zidovudine |
| BMI | Body mass index |
| CCC | Comprehensive care centre |
| CD4 | Cluster of differentiation 4 |
| CGTRH | Coast General Teaching and Referral Hospital |
| COVID-19 | Coronavirus Disease |
| CRF | Case report form |
| CTC | Care and Treatment Centre |
| DRM | Drug resistant mutation |
| DRT | Drug resistance test |
| DTG | Dolutegravir |
| eCRF | Electronic case report form |
| EFV | Efavirenz |
| EMR | Electronic medical record |
| ERC | Ethics Review Committee |
| GCLP | Good clinical laboratory practice |
| GCP | Good clinical practice |
| HIV | Human Immunodeficiency Virus |
| HIV-1 | Human Immunodeficiency Virus Type 1 |
| ICF | Participant information sheet and consent form |
| ICH | International Council for Harmonisation of Technical Requirements for Pharmaceuticals for Human Use |
| INS | Instituto Nacional de Saúde (National Institute of Health in Mozambique) |
| INSTI | Integrase strand transfer inhibitor |
| JOOTRH | Jaramogi Oginga Odinga Teaching and Referral Hospital |
| KNH | Kenyatta National Hospital |
| LPV/r | Ritonavir-boosted lopinavir |
| MUHAS | Muhimbili University of Health and Allied Sciences |
| NNRTI | Non-nucleoside reverse transcriptase inhibitor |
| NRTI | Nucleoside reverse transcriptase inhibitor |
| NVP | Nevirapine |
| PI | Protease inhibitor |
| PI/r | Ritonavir boosted protease inhibitor |
| PLWH | People living with HIV |
| RCT | Randomised control trial |
| RNA | Ribonucleic acid |
| TB | Tuberculosis |
| TDF | Tenofovir disoproxil fumarate |
| UoN | University of Nairobi |
| VL | Viral load |
| WHO | World Health Organisation |

**Investigating the optimal management of dolutegravir resistance: a multi-country cohort study**

**Study Protocol**

**Table of Contents**

[1. Introduction 3](#_Toc191546700)

[1.1 Background 3](#_Toc191546701)

[1.2 Literature Review 3](#_Toc191546702)

[1.3 Justification 5](#_Toc191546703)

[1.4 Hypothesis 6](#_Toc191546704)

[2. Study Objectives 7](#_Toc191546705)

[2.1 Primary Objective 7](#_Toc191546706)

[2.2 Secondary Objectives 7](#_Toc191546707)

[3. Overview of Study Design 8](#_Toc191546708)

[4. Study Population 9](#_Toc191546709)

[4.1 Number of Participants and Participant Selection 9](#_Toc191546710)

[4.2 Recruitment 9](#_Toc191546711)

[4.3 Inclusion Criteria 10](#_Toc191546712)

[4.4 Exclusion Criteria 10](#_Toc191546713)

[4.5 Site-Specific Recruitment 10](#_Toc191546714)

[4.6 Duration of Involvement 17](#_Toc191546715)

[4.7 Withdrawal of Participants 17](#_Toc191546716)

[5 Study Assessments and Procedures 18](#_Toc191546717)

[5.1 Time and Events Schedule 18](#_Toc191546718)

[5.2 Assessment at Each Visit 19](#_Toc191546719)

[5.3 Blood Sampling 21](#_Toc191546720)

[6 Statistical Analysis 24](#_Toc191546721)

[7 Ethical Aspects 25](#_Toc191546722)

[7.1 Ethical Considerations 25](#_Toc191546723)

[7.2 Informed Consent 25](#_Toc191546724)

[7.3 Study-specific Design Considerations 26](#_Toc191546725)

[7.4 Participant Compensation 26](#_Toc191546726)

[7.5 Privacy of Personal Data 27](#_Toc191546727)

[8 Data Handling 29](#_Toc191546728)

[8.1 Recording of Data 29](#_Toc191546729)

[8.2 Source Documentation and Study Records 29](#_Toc191546730)

[8.3 Data Management 30](#_Toc191546731)

[8.4 Storage of Data 30](#_Toc191546732)

[8.5 Quality Assurance 30](#_Toc191546733)

[9. Administrative Procedures 31](#_Toc191546734)

[9.1 Protocol Modifications 31](#_Toc191546735)

[9.2 Regulatory Notification 31](#_Toc191546736)

[9.3 Publication Policy 31](#_Toc191546737)

[9.4 Sample Shipment Processing 32](#_Toc191546738)

[10 References 33](#_Toc191546739)

[11 Protocol Signature Page 35](#_Toc191546740)

[12 Appendices 36](#_Toc191546741)

# 1. Introduction

## 1.1 Background

The majority of people living with HIV (PLWH) on first line antiretroviral therapy (ART) in low- and middle-income countries are on dolutegravir (DTG)-containing regimens [1]. Current World Health Organization (WHO) guidelines recommend that people on DTG-based first line ART with HIV viral load > 1,000 copies/mL should undergo enhanced adherence counselling and, if viral load remains > 1,000 copies/mL after 3 months, they should be switched to a protease inhibitor (PI)-based second line regimen [2]. Different countries have adopted different approaches in their guidelines. For example, Kenya recommends that after intensified adherence counselling, patients with persistent viraemia > 1,000 copies/mL should receive a drug resistance test (DRT) to guide switch and the choice of the optimal second-line regimen [3]; Mozambique, Tanzania and Lesotho recommend switch to 2 nucleoside reverse transcriptase inhibitors (NRTIs) and PI after failure of a DTG-containing first-line regimen [4, 5]; South Africa does not recommend switch from DTG or DRT for those who are on first-line DTG-containing regimens within the first 2 years of treatment, after which management is guided by possible DRT and expert opinion [6]. The switch to PI has disadvantages including higher cost, higher pill burden, less convenient administration (often should be taken with food), more potential drug-drug interactions, poorer tolerability and more long-term toxicities.

## 1.2 Literature Review

The WHO recommendation to switch to PI-based therapy is based on the untested assumptions that people failing DTG have selected for clinically relevant integrase inhibitor drug resistance mutations (DRMs) and are therefore more likely to achieve viral suppression with a change in regimen compared to remaining on a DTG-containing regimen. Clinical trial and cohort data show that DTG-based ART rarely leads to virological failure, and that the prevalence of emergent DTG DRMs is low among those with failure [7]. Cross-sectional data show varying rates of DTG DRMs among DRTs performed; however, the frequent lack of reporting of denominators on total number of people receiving DTG or total number of people with virologic failure limit the conclusions we can make about the prevalence of DTG DRMs among people with virological failure [7]. Despite low proportions of people with virologic failure and emergent DTG-associated DRMs, the absolute number of people failing DTG-based regimens has important implications for ART programs.

Emerging data from African countries have shown varying levels of integrase drug resistance mutations. Results from a recent cross-sectional survey in Malawi in children on DTG with confirmed virologic failure, found major INSTI DRM in 16.3% of 133 samples that were successfully sequenced [8], while in Mozambique, DTG resistance was found in 19.6% of 183 samples from patients with virologic failure [9]. In Kenya, surveillance samples from patients with viral non-suppression show a prevalence of up to 22.6% in ART experienced patients (on DTG as second-line or third-line regimens) and 8.3% in those on failing a first line DTG regimen [6].

Most people failing first-line DTG-based regimens without DTG-associated DRMs can be expected to re-suppress without a change in regimen if adherence and potential drug interactions are addressed, and therefore would not benefit from a change to PI-based regimen [10]. For PLWH who are failing DTG-based regimens and have developed DTG-associated DRMs, there is very limited direct evidence to guide their management. Data on pathways of DTG DRMs and their effects on in-vitro DTG susceptibility is accumulating [11], with substitutions at eight codons currently known to contribute to reduced DTG susceptibility, and thus considered “major” DTG DRMs: 66K, 92Q, 118R, 138K/A/T, 140S/A/C, 148H/R/K, 155H and 263K (Stanford HIV Drug Resistance Database version 9.6, last updated 9-Mar-2024). How the genotypic resistance patterns relate to the in-vivo virologic response to a DTG-containing ART regimen is uncertain, and multiple management strategies are currently being used including: increasing DTG to twice-daily dosing, switching from DTG to a PI, adding a PI to the DTG-based regimen, among others.

In a recent scoping review, Tao and colleagues found that major INSTI-associated DRMs clustered into four signature positions including R263K, G118R, N155H and Q148H with minimal overlap [11]. The majority of viruses had just one signature mutation, predominantly R263K. Other than G118R, the other DRMs alone were not associated with high levels of reduced DTG susceptibility and studies to determine the significance of these on clinical management are needed.

The VIKING trials assessed the efficacy of twice daily DTG plus an optimised background regimen in heavily treatment experienced patients who had previously received a first generation INSTI and had INSTI DRMs [12-15]. VIKING was a single-arm phase IIb study that initially evaluated once-daily DTG; however, poor virologic response in the initial cohort prompted a protocol change to test twice-daily DTG. Viral suppression to <50 copies/ml at week 24 was achieved by 41% of participants in the once-daily DTG cohort, and by 75% in the twice-daily DTG cohort [14]. VIKING-3 was a single-arm phase III study which further evaluated the DTG twice-daily plus optimized background therapy strategy, with 69% achieving viral suppression to <50 copies/ml by week 24 [13]. Extrapolating the VIKING series of results to the population of patients failing a first line DTG-based regimen with DTG DRMs has limitations: the VIKING participants were highly treatment experienced which may be an indicator of more extreme adherence challenges; they had resistance to at least 3 drug classes which may have synergistic effects on virologic response, and recognized INSTI DRMs at the time were different than current standards. Given that some participants in VIKING achieved viral suppression on single-dose DTG despite INSTI DRMs, it may be reasonable to test whether single-dose DTG could provide a better response in a population that is less extensively treatment experienced.

Evidence for switching from DTG to a PI is extrapolated from studies where participants failed a non-DTG first-line regimen and were treated with a PI-based second line regimen. Most relevant for our proposed study population is the NADIA trial which enrolled people failing an NNRTI plus TDF/3TC and randomized them to second line therapy of either DTG or DRV/r, plus either TDF/3TC or AZT/3TC [16]. At week 48, 92% of participants on DRV/r achieved viral suppression <400 copies/ml compared to 90.2% on DTG, a non-statistically significant difference between the arms. A limitation of extrapolating these results (and those of other second-line PI studies) to the population of patients failing a first line DTG-based regimen with DTG DRMs is that DTG-based first-line is simpler to take and better tolerated than EFV or NVP, with a higher barrier to resistance, so people failing the DTG-based regimen may be a sub-group with more barriers to adherence.

Real-world suppression rates of patients with documented DTG DRMs have been published from national program data in Malawi [17]. Among the 24 patients with DTG resistance, 18 had been on two or more regimens prior to DTG. Of 11 patients with follow-up viral load data available after implementing DRT-based regimen changes (either 2NRTI + PI/r; 2NRTI + PI/r + DTG; 2NRTI + PI/r + DTG + DTG; 2 NRTI + DTG + DTG), 9 (82%) achieved a viral load < 200 copies/mL. All participants who received double-dose DTG (4/4) achieved viral suppression, and 5/7 who received PI/r or PI/r + single-dose DTG achieved viral suppression. Of two patients who remained on DTG without a change in regimen despite DTG DRMs, 1 of them re-suppressed.

## 1.3 Justification

The WHO has recognised the role of drug resistance testing (DRT) in a treatment failure algorithm for people living with HIV receiving DTG-based treatment to minimise unnecessary switches from this regimen [2]. However, routine DRT is unlikely to be available in many resource-limited settings in the near future. The GIVE MOVE trial, in Lesotho and Tanzania, randomised children and adolescents with recent viraemia on first-line ART to the usual care arm (which consisted of a viral load-informed treatment) or to a DRT arm in which DRT and expert review informed care; they found no significant difference in the primary outcome (death, hospitalisation, new WHO stage 4 event or VL ≥50 copies/ml) between the two groups [18].

Taken together, a range of information using different viral load suppression thresholds, different approaches to DRT, and unproven extrapolation from other studies currently inform our understanding of DTG DR management. While we do not expect widespread, routine program access to DRT or TFV-DP levels, there is an opportunity to conduct a special study that, in the context of enhanced adherence monitoring and support, will provide the scientific underpinning for whether and when viremia with DTG resistance requires regimen switch – and in turn inform whether and when DRT is needed when viremia with DTG is detected through routine viral load screening.

We seek to address the gap in published data on viral suppression among people meeting criteria for virologic failure on DTG-based ART regimens without a change in regimen, and prospective data on emergence of DTG DRMs and the impact of those DRMs on suppression. We will evaluate the viral suppression and emergence of DRMs among PLWH with sustained viremia on DTG-based ART through a prospective cohort evaluation (the Ndovu Cohort Study). We will also evaluate the optimal management of people failing DTG with DRMs through a randomized clinical trial nested within this cohort study (Ndovu RCT, which will be submitted as a separate study protocol).

The data generated from these studies will inform WHO and national guidelines on management of DTG failure.

## 1.4 Hypothesis

At least 70% of participants will achieve HIV-1 RNA < 200 copies/mL within 12 months from study enrolment, with differences in suppression between sub-groups of age, sex and enrolment viral load strata.

# 2. Study Objectives

## 2.1 Primary Objective

- To assess viral suppression rate following enhanced adherence counseling among people on DTG-based ART who have viraemia (≥ 1,000 copies/mL) after at least six months on ART

## 2.2 Secondary Objectives

- - - To determine time to viral suppression to HIV-1 RNA < 200 copies/mL
    - To assess viral suppression among different age strata (1-9 years, 10-19 years, ≥20, 20-24 years, 25-34 years, 35-44 years, and ≥45 years)
    - To assess viral suppression among different viral load strata (1,000-99,999 copies/ml, ≥100,000 copies/ml)
    - To assess viral suppression based on prior PI or INSTI exposure
    - To assess viral suppression based on prior PI or INSTI failure
    - To assess viral suppression based on participant’s sex
    - To assess viral suppression based on the NRTI component of the ART regimen
    - To evaluate durability of suppression at 3 months from first VL < 200 copies/mL and after 12-24 months from enrolment
    - To quantify the incidence of treatment-emergent DRMs
    - To describe DRM patterns (DTG-associated DRMs with or without concomitant NRTI DRMs) associated with sustained non-suppression or viral rebound after suppression
    - To assess predictors of development of DTG-associated DRMs (age, other non-INSTI DRMs, VL (copies/mL) at switch to DTG, time with viremia, NRTIs used, etc.)
- To evaluate time from first detected viraemia to development of DTG-associated DRMs

# 3. Overview of Study Design

This is a multi-country observational prospective cohort study over 12 months describing HIV-1 viral suppression in people with high viral load (≥ 1000 copies/mL) after at least six months on DTG-based ART.

The study will enrol participants receiving HIV care in the participating countries and follow them at the cohort study sites (Appendix 1). As illustrated in figure 1, study visits and viral load testing will take place at enrolment and then every 3 months for up to 12 months during the active follow-up period for participants who do not achieve viral suppression < 200 copies/mL. For participants who achieve the primary outcome of HIV-1 RNA < 200 copies/mL during the active follow-up period, a repeat VL will be performed after 3 months; outcomes from routinely collected program data (viral load, loss to follow-up, death) will be collected 12-24 months from enrolment to assess durability of suppression among this group.

The schedule of visits and investigations are outlined in the Time and Events table (Section 5.1). Participants in the cohort study will also be assessed for eligibility to enrol into a randomized clinical trial on management of people who develop DRMs during the cohort study (Ndovu RCT).

**Figure 1: Ndovu Cohort Study Schema**

# 4. Study Population

## 4.1 Number of Participants and Participant Selection

The target sample size for this study is 6,600 participants. Assuming 70% of participants achieve the primary outcome of HIV-1 RNA < 200 copies/mL within 12 months from enrolment, this will provide a standard error of the estimate for viral suppression of 0.0062.

The sample size will provide a standard error of the estimate for viral suppression for pre-specified sub-group analysis as follows:

- Age categories, assuming 6% aged 1-9 years, 18% 10-19 years and 76% ≥20 years, we will have standard errors of 0.025, 0.015 and 0.0071 respectively.
- Gender strata, assuming 63.5% females and 36.5% males, we will have standard errors of 0.0076 and 0.010 respectively.

The formula used to calculate the standard error of a proportion is:

$$S.E.=\sqrt{\left( \frac{(p(1-p))}{n} \right)}$$

Participants who develop DTG-associated DRMs during cohort follow-up will be assessed for eligibility into a clinical trial assessing the optimal management of people who develop drug resistance to DTG-based ART (Ndovu RCT). Assuming 5.5% of the cohort participants will develop DTG DRMs, the cohort sample size will allow full enrolment into the Ndovu RCT. Given the uncertainty in DRM estimates, once 1,000 participants have been enrolled into the cohort we will refine the estimated proportion of participants with DTG DRMs and adjust the sample size calculation if needed to fully enrol the Ndovu RCT. Any proposal to adjust the sample size will be submitted as a protocol amendment to the ERCs.

## 4.2 Recruitment

Participants will be recruited without advertisement from the pool of patients receiving routine outpatient HIV care at the study sites and at surrounding HIV clinics. Pre-screening using national viral load databases and study site electronic medical records will identify potential participants to invite for screening. If potential participants do not have a viral load performed within the preceding 3 months then facility staff will be requested by the National HIV Program to invite them for follow-up viral load testing. Pre-screening will also include a review of national DRT databases to identify all patients with at least one major DTG-associated DRM, followed by a chart review to determine if their most recent viral load is ≥1,000 copies/mL and that they are still on a DTG-based ART regimen without concomitant NNRTI or PI, in which case they will be invited for screening at the participating study sites. Those who provide informed consent at screening will be assessed for eligibility.

## 4.3 Inclusion Criteria

Participants must satisfy all of the following criteria to be enrolled in the study:

- Able and willing to provide informed consent (assent as appropriate and legal guardian consent if < 18 years)
- Age ≥ 1 years
- Documented HIV-1 infection as confirmed by national HIV testing standards at the respective study sites
- On a DTG-based ART regimen for at least six months
- Most recent HIV-1 RNA ≥ 1,000 copies/mL within 3 months prior to enrolment, taken after at least 6 months on current ART regimen

## 4.4 Exclusion Criteria

Potential participants who meet any of the following criteria will be excluded from participating in the study:

- Any reason which, in the investigator’s opinion, will significantly prevent collection of viral load levels such as relocation to another area outside of the trial sites or imminent death
- Concomitant NNRTI or PI while on DTG

## 4.5 Site-Specific Recruitment

Participants will be enrolled from 8 central study sites in Kenya, 1 central study site in Tanzania (enrolling from surrounding care and treatment centres in Dar es Salaam), 6 study sites in Mozambique and 5 study sites in Lesotho. For each country, more study sites may be included based on the actual numbers. The cohort sample size is estimated to be 6,600 but is not fixed because enrollment will continue until the nested RCT reaches its pre-specified sample size. Table 1 summarises the number of sites and enrolment targets for each country:

**Table 1: Sites and Enrolment Targets by Country**

| **Country** | **Number of sites – Cohort*** | **Number of sites - RCT** | **Proposed cohort enrolment target** | **Proposed RCT enrolment target** |
| --- | --- | --- | --- | --- |
| Kenya | Sites in Nairobi, Kisumu, Mombasa, Siaya, Kiambu, Bungoma, Trans Zoia and Homa Bay Counties* | 3 - JOOTRH, KNH, Bomu Hospital | 2,400 | 144  (132 adults + 12 children) |
| Tanzania | 1 - Clinical trial unit (CTU) at MUHAS enrolling from CTCs in Dar es Salaam | 1 - CTU | 900 | 56  (50 adults + 6 children) |
| Mozambique | 3 sites in Maputo and 3 sites in Sofala | 2 - in Maputo and 1 in Sofala | 2,400 | 144  (132 adults + 12 children) |
| Lesotho | 5 sites: Butha-Buthe, Mokhotlong, Motebang, Berea, Senkatana | 2 | 900 | 48 |
| Total |  |  | **6,600** | **392** |

* Sites are listed in appendix 1

Where a country fails to meet the target numbers, other countries may enroll additional numbers.

**4.5.1 Country-Specific Site Description and Recruitment Strategy**

Table 2 summarises the country-specific guidelines and recruitment strategies for this study.

**Table 2: Country-Specific Site Description and Recruitment Strategy**

|  | Kenya | Mozambique | Tanzania | Lesotho |
| --- | --- | --- | --- | --- |
| Preferred first line regimen for adults and adolescents | Children and adolescents <30 kg: ABC/3TC+DTG; Adults and adolescents ≥30 kg: TDF/3TC/DTG | Children and adolescents <30 kg: ABC/3TC+DTG; Adults and adolescents ≥30 kg: TDF/3TC/DTG | Infants and children <20 kg: ABC+3TC+LPV/r; Children and adolescents ≥20 kg: ABC+3TC+DTG; Adults and adolescents ≥30 kg: TDF+3TC+DTG | Children and adolescents <35kg: ABC/3TC+DTG; Adults and adolescents ≥35kg: TDF/3TC+DTG |
| VL testing as per current national guidelines* | HIV VL testing is performed 3 months after ART initiation and 6 to 12 monthly thereafter depending on age. If VL ≥200 copies/mL, enhanced adherence counselling and repeat VL after 3 months of good adherence. If repeat VL ≥200 copies/mL, consult regional or national technical working group. A DRT may be conducted if repeat VL ≥1,000 copies/mL | HIV VL testing is performed 6 months of ART initiation and annually thereafter. If VL ≥1,000 copies/mL, enhanced adherence counselling and repeat VL done 3 months. If repeat VL ≥1,000 copies/mL, consult the Therapeutic Committee. If VL Is ≥50 and <1,000copies/mL, conduct enhanced adherence counselling and repeat VL after 12 months | HIV VL testing is performed 6 months of ART initiation and annually thereafter. If VL is ≥1,000 copies/mL enhanced adherence counselling is done for 3 consecutive months followed by repeat VL; if the repeat VL is ≥1,000 copies/mL, switch to the default second line PI-based treatment | HIV VL testing is performed 6 months of ART initiation and 6-12 monthly thereafter depending on population. PLWH with VL >50 copies/mL undergo enhanced adherence counselling and repeat VL done 3 months with adherence issues addressed. If repeat VL >50 copies/mL, consult ART advisory committee; switch of ART if repeat VL ≥1,000 copies/mL |
| Recommended switch regimen | DRT-based second-line | AZT+3TC+LPV/r or ATV/r | ABC+3TC+ATV/r or LPV/r | ABC/3TC+LPV/r or ATV/r or TDF/3TC+ATV/r or LPV/r if ≥35kg |
| Location of study sites | Nairobi, Kisumu, Kiambu, Trans Nzoia, Bungoma, Homa Bay, Siaya and Mombasa Counties | Maputo and Sofala Provinces | Muhimbili University of Health and Allied Sciences (MUHAS) Clinical Trial Unit located within the Muhimbili National Hospital’s premises | Butha-Buthe, Mokhotlong, Leribe, Teyateyaneng, and Maseru Districts |
| Approximate number of patients with VL ≥1,000 copies/mL | About 6,940 in the last 12 months | About 2,400 in the last 6 months in the 6 cohort sites, with several more in the surrounding facilities | About 85 at Muhimbili National Hospital and about 3,484 in the 5 municipalities within Dar es Salaam | About 1,000 in the last 12 months |
| Where and how recruitment activities will be taking place | As listed in appendix 1, 8 hub sites will be established as the base sites for study teams in the 5 counties with participants enrolled from both the hub and spoke sites until the enrolment target is met. Follow-up visits will be conducted at the site where the participant is recruited | Plan to recruit from the 6 base cohort sites; if the full cohort cannot be enrolled from these sites, then surrounding facilities will be invited to participate with recruitment and follow-up being done at the base sites | Will recruit from Muhimbili National Hospital and the 205 care and treatment centres (CTCs) in Dar es Salaam | Recruitment will be done at the study sites: Butha-Buthe District Hospital, Mokhotlong Hospital, Motebang Hospital, Berea Hospital, and Senkatana Hospitals. For Butha-Buthe and Mokhtolong districts, the identified hospitals will be established as study sites with recruitment of additional participants from the surrounding health facilities. |
| Pre-screening strategy | Potential participants will be identified by working with the national HIV program and implementing partners, using the national HIV medical record system (Kenya EMR), to generate line lists of all patients at the recruitment sites who are on DTG-based ART for at least 6 months with their most recent VL ≥ 1,000 copies/mL | Eligible participants will initially be flagged on the patient file by the study site nurse. The study nurses will verify their next clinic visit date or will call to schedule a visit if a phone number is available. The site nurse will contact the research assistant about the visits | The study team will work with Management and Development for Health (MDH) to develop a line list of CTCs indicating individuals on DTG-based therapy with VL ≥1,000 copies/mL. Investigators at MUHAS will link with the CTC managers, starting with CTCs with the highest numbers on the line list, to identify and refer these individuals to the study team members at the MUHAS clinical trial unit. The study team will brief the potential participant about the study and schedule an appointment to visit to the clinical trial unit for detailed study information and consenting. | The study team will review the VICONEL study database and develop a line list of those with a VL of ≥1,000 copies/mL in the preceding 12 months. For study sites that have not been connected to the VICONEL database, the study team will identify patients who qualify for cohort inclusion through the patient files at the study sites. The study team will then verify the next clinic visit for all potential participants or schedule a timely visit if none had been planned. |
| Cohort recruitment and follow-up | All patients who attend a screening visit and provide informed consent will be assessed for eligibility; those who meet eligibility criteria will be enrolled in the cohort study and scheduled for a follow-up visit in 3 months, which will take place at the same site as their recruitment visit | When an eligible participant arrives at the health facility, the research assistant will proceed with the informed consent and the data collection. If the participant accepts to be included in the study, a study ID will be attributed and blood collection will be done. The site nurse will be responsible for proceeding with blood collection and monitoring the next visits. | All consenting individuals will be screened and assessed for eligibility at the clinical trial unit. Participants fulfilling the eligibility criteria will be enrolled in the cohort and followed up according to the study protocol at the clinical trial unit. | Identified possible participants who come to the clinic for a follow-up assessment will be approached by the study team to inform them about the study and get their consent for participation. People who consented and meet the eligibility criteria will be enrolled in the cohort study. Data collection and follow-up will be done according to the study protocol. This can be done at both the central study sites as well as satellite sites. |
| RCT recruitment and follow-up | Potential participants will be assessed for RCT eligibility at their cohort follow-up site, along with their interest in participating in the RCT. Those who meet eligibility criteria and consent to participation in the RCT will be invited to an RCT screening visit at one of the 3 RCT sites (KNH, JOOTRH or Bomu Hospital) | For the Ndovu RCT, eligible participants will be referred to the clinical trial units in Maputo and Beira for consenting, eligilibity assessment and follow-up | Both the Ndovu Cohort Study and RCT will be conducted at the same site. Participants from the cohort study who may be eligible for the RCT will be informed of the RCT and referred to the RCT screening room for consenting and eligibility assessment. If they do not consent to the RCT or are not eligible, they will continue follow-up in the cohort study at the same site | Cohort participants who are eligible for participation in the RCT will be referred to the study teams at the central study sites. Once consent has been received for participation in the RCT, eligible participants will be randomized. They will be treated and followed up according to protocol. All follow-up visits for the RCT will be done at the central study sites. |
| Viral load testing and DRT | VL testing will be conducted at the National Public Health Laboratory (NPHL) in Nairobi and its regional testing facilities in Kisumu, Migori and Mombasa; the Roche COBAS 8800, Abbott m2000 rt and Abbott Alinity testing platforms will be used. DRT will be conducted at the NPHL | VL testing within Maputo Province will be done at the Biotechnology and Genetics Laboratory in INS while for Beira Province VLs will be processed by the Molecular Biology Laboratory in Ponta Gea; testing will be done on plasma samples using the Roche COBAS 6800 and Abbott Allinity platforms. DRT will be done at the INS reference laboratory | Both VL and DRT samples will be processed at the Temeke Specialized Laboratory. For VL testing, plasma samples will be used and testing done on the Roche COBAS 6800 and 8800 platforms. | VL testing will be done at the respective site laboratories on the Roche COBAS 4800 platform using plasma samples. Samples for DRT will be processed at a referral laboratory in South Africa (Lancet Laboratories and PathCare) |

* The national AIDS control programs in each country have been engaged to allow this study to proceed as per this protocol despite deviation from the current national guidelines

## 4.6 Duration of Involvement

Participants will be engaged in active cohort follow up for up to 12 months and will be consented for use of their routinely collected viral load and clinical outcomes data from 12 to 24 months after enrolment.

## 4.7 Withdrawal of Participants

A participant is free to withdraw from the study at any time.

# 5 Study Assessments and Procedures

## 5.1 Time and Events Schedule

**Table 3: Time and Events Table**

| **Cohort**  Procedure | Pre-screening | Months from enrolment | | | | |
| --- | --- | --- | --- | --- | --- | --- |
|  |  | Screening / Enrollment (Day 1)^1^ | 3 | 6 | 9 | 12 |
| **Clinical and other assessments** | | | | | | |
| Written informed consent |  | x |  |  |  |  |
| Inclusion/exclusion criteria |  | x |  |  |  |  |
| ART history | x | x |  |  |  |  |
| Medical history (past and current) |  | x |  |  |  |  |
| Concomitant medication |  | x | x | x | x | x |
| Smoking, alcohol and other drug use history |  | x | x | x | x | x |
| Vital signs |  | x | x | x | x | x |
| Physical examination |  | x | x | x | x | x |
| Height/Length^2^ |  | x | x | x | x | x |
| Weight |  | x | x | x | x | x |
| Mid-upper arm circumference (MUAC)^3^ |  | x | x | x | x | x |
| Head circumference^4^ |  | x | x | x | x | x |
| Clinical screening for TB |  | x | x | x | x | x |
| Clinical assessment for opportunistic infections |  | x | x | x | x | x |
| Enhanced adherence counselling |  | x | x | x | x | x |
| Dispensing of medications |  | x | x | x | x | x |
| Pill counts and Proportion of Days Covered |  | x | x | x | x | x |
| **Laboratory investigations** | | | | | | |
| HIV-1 RNA viral load^5^ | x^6^ |  | x | x | x | x |
| Drug resistance test^7^ |  |  | x | x | x | x |
| Urine pregnancy test^8^ |  | x | x | x | x | x |
| Stored plasma sample^9^ |  | x | x | x | x | x |
| - 1. Enrollment within 3 months of pre-screening viral load   2. Height for adults will be measured during the screening visit only. Length/height will be measured during each visit for participants aged 1 to 18 years old   3. MUAC will be measured for children aged 1 to 5 years old   4. Head circumference will be measured for children under the age of 5 years   5. For participants achieving HIV-1 RNA < 200 copies/mL, outcomes from routinely collected program data (mortality, loss to follow up, and viral load) will be documented 12 to 24 months after enrolment   6. Pre-screening VL results from within 3 months prior to enrolment will be documented   7. Drug resistance testing will be performed for all participants with VL ≥ 200 copies/mL at months 3, 6, 9 and 12   8. For women of child bearing potential   9. Plasma will be stored for drug resistance testing if VL is ≥200 copies/mL | | | | | | |

## 5.2 Assessment at Each Visit

The schedule of assessments is summarized in the Time and Events table (table 2).

Written informed consent will be obtained at the start of the screening/enrolment visit before engaging in any study related evaluations and procedures.

All participants will complete the screening assessment of eligibility criteria on the same date as informed consent is obtained; if eligibility criteria are met, they will be enrolled into the study on the same day. The first follow-up visit will take place at month 3 for all participants. Additional follow up visits will take place at months 6, 9 and 12 for participants who have not achieved HIV-1 RNA < 200 copies/mL. Participants who achieve HIV-1 RNA < 200 copies/mL at months 3, 6 or 9 will have an additional follow-up visit after 3 months and if HIV-1 RNA is still < 200 copies/mL will exit from active follow-up; those whose follow-up HIV-1 RNA is ≥ 200 copies/mL will continue with active follow up in 3 months and further follow up will be determined by the subsequent viral load results.

All participants will undergo enhanced adherence counselling sessions as well as an assessment of other reasons for viremia, such as drug-drug interactions, incorrect dosing, or poor absorption starting at enrollment. Protocol-specified enhanced adherence counseling and assessment/management of other causes of viremia will continue for at least 3 sessions and will be carried out in person at the clinic and via phone calls where feasible. Additional enhanced adherence counseling will be performed for all participants with HIV-1 RNA ≥ 200 copies/mL at follow up visits.

Dispensing of medication will be done at the site pharmacy as per national protocols. This will follow national guidelines with the participant’s medication refill tallying with the timing and frequency of visits in-between the protocol-defined study visits based on adherence assessment and viral load. Adherence will be assessed by use of pill counts and the proportion of days covered (PDC).

HIV-1 RNA will be measured at enrolment and months 3, 6, 9 and 12.

Any cohort participant whose HIV-1 RNA is ≥ 200 copies/mL at any follow up visit will have a reflex DRT performed. If DRT shows no major DTG DRM or is unsuccessful in sequencing the INSTI region then they will have additional enhanced adherence counseling and will continue on their DTG-based ART until the next follow-up viral load 3 months later, after which further action will be determined by the repeat viral load and DRT results.

If DRT shows ≥1 major DTG-associated DRM based on pre-enrollment historical DRT results or any study DRT, then the participant will be assessed for enrolment into the RCT; those not eligible for the RCT will have management determined by a protocol-defined management algorithm and remain in the cohort. Protocols for management of this subgroup of patients will be developed with the various national programs and based on national guidelines as well as availability of various antiretroviral agents.

For participants who achieve HIV-1 RNA < 200 copies/mL, outcomes from routinely collected program data (viral load, loss to follow-up, death) will be documented 12 to 24 months after enrolment.

Follow up visits will take place within +/- 14 days of the specified visit schedule. Participants will be contacted by phone (phone call or text message) 24-48 hours before each appointment, and if they do not attend a scheduled visit they will be contacted by phone the following day. Additional/unscheduled visits are allowed as needed for clinical indications such as intercurrent illnesses, management of comorbidities, or as desired by the participant.

Clinical measurements following the Time and Events Schedule (Section 5.1) will include:

- Height: Standing height will be measured once to the nearest 0.5 cm barefoot, the back square against the wall tape, eyes looking straight ahead, with a set square resting on the scalp and against the wall (WHO, 1995)
- Length: For children who are less than 2 years old or older and cannot stand, length will be measured to the nearest 0.5 cm with the child lying on a measuring board with a sliding foot piece
- Weight: Weight will be assessed once to the nearest 100 grams using a lever balance, barefoot, in light garments (WHO, 1995)
- Body Mass Index: The body mass index (BMI) will be calculated using the World Health Organization (WHO) formula as weight (in kilograms) divided by height (in meters) squared (WHO, 1995)
- Mid-upper arm circumference (MUAC): MUAC will be measured for children aged 1 to 5 years old in centimetres on a straight left arm (right if it is the non-dominant arm) midway between the tip of the shoulder and tip of the elbow using a MUAC tape
- Head circumference: will be measured for children aged 1 to 5 years old in centimetres using a flexible tape placed just above the eyebrows, above the pinnae and around the occipital prominence at the back of the head. The tape will be pulled gently to compress the hair and the circumference will be measured to the nearest 0.1cm
- Blood Pressure: Blood pressure will be measured using an automated blood pressure machine with the appropriately sized adult and pediatric blood pressure cuffs around the mid arm with the participant sitting upright, both feet flat on the floor, with the back and arm supported (WHO, 1999)

For girls or women of child bearing potential (WOCBP), a urine sample will be collected during each visit for a point of care pregnancy test. WOCBP refers to female participants following menarche until menopause (no menses for 12 months without alternative medical cause) unless permanently sterile (undergone bilateral tubal ligation or hysterectomy).

## 5.3 Blood Sampling

Blood will be collected for laboratory investigations as outlined in the Time and Events Table (Section 5.1). Blood will be drawn from the antecubital fossa or any other appropriate site and collected in vacutainers. A total of up to 48 mL of blood will be collected from each study participant as follows:

- Enrollment: 8 mL for adolescents (10 years old and above) and adults, and 6 mL for children less than 10 years old) of blood collected in one or two vacutainer tubes
- Month 3: up to 10 mL of blood collected in one or two vacutainer tubes
- Month 6: up to 10 mL of blood collected in one or two vacutainer tubes
- Month 9: up to 10 mL of blood collected in one or two vacutainer tubes
- Month 12: up to 10 mL of blood collected in one or two vacutainer tubes

All the standard procedures for performing these tests will be maintained to ensure results are of highest quality.

**5.3.1 Sample Processing in Kenya**

All blood samples collected from clinics across Kenya will undergo processing at the Kenya National Public Health Laboratory, including its associated regional testing facilities: KEMRI CGHR, KNH CCC, Kenyatta University Hospital, KEMRI Alupe, Migori County Referral Hospital, KEMRI Walter Reed, and AMPATH Care Laboratory. These facilities are fully ISO 15189 accredited, adhere to stringent internal and external quality control standards including a common inter-laboratory comparison scheme, and are equipped to conduct all necessary tests for the study with a result turnaround time of ten working days. Samples will be transported to the testing laboratory following the guidelines outlined in the study's standard operating procedures manual ensuring that the sample chain of custody is maintained. Any leftover samples will be destroyed locally after analysis.

At each time point a sample will be stored for subsequent drug resistance testing if the viral load is ≥200 copies/ml. These sample will be transported on ice to the storage laboratories located at the National Genomics Laboratory at the NPHL, and the MIDR laboratory at the University of Nairobi for processing and storage of plasma aliquots at -80 ^0^C. Some of the aliquots will be released for DRT at the National Genomics Laboratory if the viral load at that timepoint is ≥200 copies/ml.

The Cobas 8800, Abbott m2000rt, Abbott Alinity, Hologic testing platforms, and Cepheid GeneXpert will be utilized to measure HIV viral load by assessing HIV-1 RNA levels. Any samples showing unsuppressed viral loads above or equal to 1000 copies/ml and those with low-level viremia above 200-999 copies/mL will be transferred to the National Public Health Institute Genomics and Molecular Surveillance Laboratory. There, HIV drug resistance testing will focus on the entire Pol region using the Sanger 3730xl platform. Additionally, the Illumina MiSeq will be employed for whole-genome sequencing.

**5.3.2 Sample Processing in Mozambique**

All samples (plasma or plasma separation cards) collected within Maputo Province will be tested at the reference laboratory (Biotechnology and Genetics Laboratory of INS) located in Marracuene using COBAS 6800 (Roche Diagnostics). The reference laboratory is fully accredited (ISO 15189), uses internal controls, and participates in an external quality assurance control program for HIV VL testing.

All the samples collected in Beira Province will be processed by the Molecular Biology Laboratory in Ponta Gea which uses Allinity (Abbott) to test all the HIV VL samples. This laboratory also uses internal quality control and participates in an external quality control program.

Samples collected at the study site will be transported in an icebox to the VL testing laboratory at between 2 and 8°C if VL testing can be performed within 24 hours. Otherwise, plasma will be separated and stored at -70 to -80°C until VL testing is performed. After VL testing, if the VL is ≥200 copies/mL, the remnant plasma should be aliquoted in a separated cryovial-tube within 24 hours at 2 – 8°C and then frozen immediately at -70 to -80°C until DRT testing day at INS.

For samples that have VL testing done in Sofala, the remnant and aliquoted plasma after VL testing will be frozen at -70 to -80°C until shipping day to INS for DRT or storage. When shipping, the frozen aliquots will be packed with ice packs in a cryobox. After reception at INS and registration on the study database, the plasma specimens will be stored at -70°C, if not processed immediately. A mapping of all samples will be done, indicating its location in the cryobox, cryobox number, freezer number, and position in the freezer.

At INS, aliquots for DRT will be processed using Oxford Nanopore GridION (https://nanoporetech.com), which is a portable sequencer that identifies DNA bases by measuring the changes in electrical conductivity generated as DNA strands pass through a biological pore. A modified SARS-CoV-2 MIDNIGHT sequencing protocol for HIVDR using GridIon from Oxford Nanopore Technology will be used. The MIDNIGHT protocol is a modification of the ARTIC amplicon V3 sequencing protocol for MinION for nCoV-2019 developed by Josh Quick.

**5.3.3 Sample Processing in Tanzania**

All samples collected at the MUHAS clinical trial unit will be processed at the Temeke Specialized Laboratory which is internationally accredited with ISO15189; 2022. The laboratory can process up to 4,200 viral load samples and 56 for drug resistance within 24 hours using Cobas 6800 and 8800 machines for viral load and Genetic analyser systems 3500 XL Applied Biosystem for drug resistance testing.

Whole blood samples from the Clinical Trial Unit at Muhimbili University of Health and Allied Sciences (MUHAS) will be transported to Temeke Specialized Laboratory within six hours of collection. Upon arrival, the samples will be centrifuged to separate plasma for VL testing. If VL testing is not performed within 24 hours, the plasma samples will be stored at -80^0^C freezer in cryotubes until testing. Samples with a VL of ≥ 200 copies/ml will undergo drug resistance testing at the same laboratory using Applied Biosystems™ 3500 XL Genetic Analyzer (Thermo Fisher Scientific, Germany).

**5.3.4 Sample Processing in Lesotho**

In Lesotho, blood samples will be analysed by the respective site laboratories. The laboratories comply with national and international regulatory standards. In case viral load testing is not available on site, samples will be transported to the nearest testing laboratory by a dedicated motorbike transport service. Viral load testing will be done on the Roche COBAS 4800 platform.

Samples for DRT will be transported and analysed in South Africa given that there is currently no in-country capacity for resistance testing.

# 6 Statistical Analysis

We will describe the characteristics of the cohort using counts and proportions for categorical variables and means and standard deviations or medians and interquartile ranges for continuous variables.

We will assess viral suppression rate following enhanced adherence interventions among people on DTG-based ART who have sustained viremia (≥ 1,000 copies/mL) after at least six months on ART using generalized linear regression model.

We will estimate overall viral suppression, defined as HIV RNA < 200 copies/mL, in the cohort at 6 and 12 months, using a generalized linear regression model with binomial distribution.

Durability of suppression at 3 months from first VL < 200 copies/mL and after 12-24 months from first VL < 200 copies/mL will be summarized using counts and proportions.

We will also use the generalized linear regression model to estimate differences in the proportions with viral suppression:

- among different age strata (1-9 years, 10-19 years, ≥20 years)
- among different VL strata (200-999 copies/ml, ≥1000 copies/ml)
- between participant sex at birth
- suppression based on NRTIs

Logistic regression models will be used to assess predictors of achieving suppression, development of DTG-associated DRMs (age, other DRMs, VL at switch to DTG, time with viremia, etc.) and development of opportunistic infections. Proportional hazards regression will be used to compare time from enrolment to development of DTG-associated DRMs between treatment groups and time to VL suppression defined as HIV RNA < 200 copies/mL.

# 7 Ethical Aspects

## 7.1 Ethical Considerations

The protocol for this study, participant information and consent form (ICF), data collection tools and information about compensation available to participants, along with any other documentation required to fulfil Ethics Review Committees (ERC) obligations, will be submitted to the ERCs covering each study site as follows:

- Kenya:
  - Kenyatta National Hospital and University of Nairobi Ethics Review Committee (KNH-UoN ERC)
  - Jaramogi Oginga Odinga Teaching and Referral Hospital’s Institutional Scientific and Ethical Review Committee (JOOTRH ISERC)
- Tanzania:
  - Muhimbili University of Health and Allied Sciences Research Ethics Committee
  - National Health Research Ethics Committee (NatHREC)
- Mozambique:
  - INS approval from the INS Scientific and Technical Review and the INS Institutional Review Board (CIBS-INS)
  - Comité Nacional de Bioética para Saúde (CNBS) - National Ethics Committee
- Lesotho:
  - National Health Research Ethics Committee

Any changes will be submitted as numbered and dated protocol amendments in accordance with ERC regulations.

## 7.2 Informed Consent

Potential participants ≥ 18 years will be provided with a verbal explanation of the nature of the study and a written participant information sheet provided.

The participant and/or guardian will be given adequate time for questions and clarifications before agreeing to participate. The investigator will explain the aims, methods, objectives and potential hazards of the study, as well as clarify to the participant that they are free to withdraw from the study at any time point for any reason without any detriment. In Kenya, informed consent will be administered in English, Kiswahili or Dholuo; while in Tanzania, this will be done in Kiswahili or English; in Mozambique, Portuguese; and in Lesotho, English and Sesotho languages will be used for informed consenting. Written informed consent will be obtained before the initiation of any study related evaluations and procedures. Any potential participant who is illiterate will choose an impartial witness (who is not a study team member); the witness must take part in the entire informed consent process, and the witness will counter-sign beside the participant’s thumbprint mark of agreement.

Written informed consent for participants aged 1 to 12 years will be provided by their caregivers or parents.

Participants aged 12 to 17 years who are accompanied by the caregivers will provide assent in addition to the written informed consent provided by their caregivers. This written assent only applies to adolescents aged 12 to 17 years who have been disclosed to about their HIV status and will be provided after being taken through what the study procedures entail in a simpler language that they are able to easily understand. Where disclosure has not been done, we will get caregiver consent for enrollment and work with the caregiver and child to support disclosure. They will sign or write initials against a thumbprint on an adolescent assent form. Their parents will have provided a written informed consent.

We request for waiver of parental consent for participants aged 15 to 17 years and unaccompanied by their caregivers. This study is a low risk study that carries minimal risk to study participants, and it does not deviate from the standard care that should be offered to an adolescent living with HIV with a high viral load.

The Participant Information Sheets and Informed Consent Forms are attached in Appendix 4 in the languages relevant to each country, which must be approved by the ERCs prior to use.

## 7.3 Study-specific Design Considerations

Risks from the study procedures and treatments, as well as the methods to reduce those risks are described in detail below.

1. The only invasive procedure we propose is phlebotomy. Phlebotomy will be performed by trained professional phlebotomists, clinicians or nurses engaged by the study or by contracted study laboratories
2. Frequent and longer visits to the health facility could increase the risk of exposure to COVID-19 and other infectious diseases. The study will make every effort to minimize this by following all recommended precautions such as use of face masks, gloves and frequent cleaning of hands, ensuring that there is adequate space between people in the waiting areas, frequent and thorough cleaning of the clinic, minimizing contact time, and ensuring that any staff in the clinic who has any symptoms is not at work and receives treatment promptly.

## 7.4 Participant Compensation

Participants will be offered compensation for transportation costs associated with attending study visits, paid in local currency for each study site.

In Kenya, the level of reimbursement will be dependent on distance travelled to and from the clinic. If one is coming to the clinic from within a 15km radius of the study site, they will be reimbursed Kenya Shilling (KES) 400/= per visit. If coming from 15 to 25km of the study site, they will be reimbursed KES 600/= per visit. If coming from further away or if they require additional arrangements to attend a clinic visit e.g. a patient escort, they will be compensated based on the prevailing public transport rates and any costs related to the visit. All payments will be made to the participant at the end of the visit via mobile money transfer (MPESA). These rates will be reviewed regularly in the course of the study to cater for changes in transportation costs.

For Mozambique, 3.3 USD will be given to participants during each study visit to cater for travel and additional time needed at the clinic during study visits. This will be paid in cash.

In Lesotho, participants will receive travel reimbursements based on the distance travelled and road conditions. Reimbursements will range from 50 Lesotho Loti (LSL) for local residents to 200 LSL for those traveling longer distances. If travel to the facility requires a four-wheel-drive or multiple connections, higher costs will be reimbursed accordingly. Payments will be made via mobile money transfer (MPESA) or in cash for those without a phone.

In Tanzania, participants enrolled in Dar es Salaam will be compensated a total of 20,000 Tanzanian shillings for transport to and from the clinic and time spent at the clinic. This amount has been used in previous clinical trials and has been approved by the local community advisory board and the ethics committees. This will be paid in cash after the visit is complete.

Transport rates may be adjusted if local transportation costs change significantly during the study period.

## 7.5 Privacy of Personal Data

Only personal data required to investigate the primary and secondary objectives will be collected from the study participants. Data will be collected and handled with safeguards to maintain confidentiality and in conformity with data privacy and protection laws and regulations.

The investigators will ensure that the personal data will be:

- Processed fairly and lawfully
- Collected for specified, explicit, and legitimate purposes and not further processed in a way incompatible with these purposes
- Adequate, relevant, and not excessive in relation to said purposes
- Accurate and, where necessary, kept current

Written consent for the processing of personal data will be obtained from the participant before collection of data. The participant has the right to access their personal data through the investigator and request alteration of any data that is inaccurate or incomplete. Adequate technical and organizational processes will be in place to prevent unauthorized access, inappropriate disclosures, accidental or unlawful destruction, or accidental loss or alteration. Investigators and study team members with access to personal data will keep the identity and participant information confidential.

Study staff will also endeavour to maintain privacy and confidentiality in all interactions with study participants. Any contacts via phone will be done without identifying the study participant’s HIV status or that they are participating in a research study to other individuals answering the phone.

Specific measures to ensure secure storage of data will be implemented. All study data will be entered in electronic CRFs on password- or biometric-protected tablets or laptops. Only study staff will have access to these tablets or laptops. Personally identifiable data will be stored distinctly separate from clinical data in password-protected and encrypted files. Any time paper-based CRFs are required they will be locked in a separate filing cabinet only accessible to the study staff. Analytic datasets will not contain personally identifiable information, and in these datasets, study participants will be identified only by a coded study identifier. The key to the coded study identifier will be stored in a password-protected and encrypted file.

# 8 Data Handling

All study data will be handled in compliance with local data protection laws and regulatory requirements, and in-keeping with Good Clinical Practice (GCP) standards.

Participant data collected during the study will be logged in study-specific electronic case report forms (eCRFs). The data management system deployed will have appropriate security measures to protect the data from unauthorized access, accidental loss, and alteration. Any data breaches will be reported to the ERCs and regulatory authorities.

To maintain confidentiality, participant data will be identified only by protocol identifier, site identifier, participant identifier and clinic identifier. During consenting, participants will be informed that the data collected during this study will be used as described in the ICF and will only be accessed by trained, authorized and contractually bound persons including the investigators, trial monitors, ERC members, and inspectors from regulatory authorities.

## 8.1 Recording of Data

All study data will be logged in the appropriate eCRFs on REDCap by trained and authorized members of the study team. Such authorization will be given in writing by the Chief Investigator or as delegated through each Country Principal Investigator before the conduct of any study related activities. A delegation of authority log identifying who can enter data and/or sign off a CRF will be kept by the Chief Investigator and each Country Principal Investigator.

In case the eCRF is inaccessible, a paper CRF will be availed for use and will be scanned to produce an electronic copy within 24 hours. The eCRF will be kept updated by entering data within one week of any paper-based data collection.

## 8.2 Source Documentation and Study Records

The study’s data management standard operating procedures list all source documents. These substantiate that the participant actually exists and assure the authenticity of the data on the eCRFs.

The participant’s recruitment date and a study identifier will be logged in their study records. These will be documented alongside the following data elements: confirmation of written consent, the participant’s clinical status, study visit date, concomitant medications, copies of all relevant reports and laboratory tests and comments on reports.

The investigator, trial monitor and delegated staff will regularly check data entered on the eCRFs against source data to ensure that the data is complete and accurate. Monitoring will also ensure that the study is being conducted in compliance with the protocol and regulatory requirements, and that the participants’ safety and rights are being protected.

## 8.3 Data Management

Source data will be securely kept in appropriately labeled binders in the study sites for the duration of the study.

Data entered on eCRFs will be kept on a secure network drive at the University of Nairobi and only accessible to authorized personnel of the data management team and the study monitor. A log of authorized personnel will be kept by the Chief Investigator and each Country Principal Investigator.

## 8.4 Storage of Data

At the end of the study, participant records and other study documentation will be stored by the Country Principal Investigator as per GCP standards and local regulatory requirements. The investigator will put in place processes to prevent their accidental or premature erasure, destruction or disposal. In the event the Chief or Principal Investigator retires, relocates, or for other reasons withdraws from the duty of being in-charge of the study records, custody will be shifted to a co-investigator who will agree to take this responsibility.

## 8.5 Quality Assurance

Study-specific eCRF completion guidelines will be developed and used to guide study staff in data entry and review. The eCRFs will have in-built data quality checks to ensure good quality data at the time of data entry. In order to ensure the accuracy and reliability of data, all eCRFs will be completed immediately by the study staff conducting the relevant procedures, unless this would pose a hazard to the study participant. When paper forms are used, they will be scanned within 24 hours and entered into the eCRF within one week.

Regular data quality checks will be done by delegated study staff. The reason for any change in data will be documented by the user and verified by one of the data management study staff. An electronic log detailing all data entered, a track of changes made, details of the user(s) entering the specific data, and time stamps will be maintained.

Written Standard Operating Procedures will be followed for all study activities to make sure the study is carried out and data are generated, recorded and reported in compliance with the protocol, GCP and the appropriate regulatory requirements.

All investigators and study personnel will maintain up-to-date GCP certification.

# 9. Administrative Procedures

## 9.1 Protocol Modifications

This protocol will not be modified without approval of a formal amendment. Protocol amendments will not be implemented before ERC approval, or when the relevant competent authority has raised any grounds for non-acceptance, except when necessary to address immediate dangers to participants. In the latter case, the modifications will be promptly presented to the ERCs. When the alteration(s) relate only to logistic or administrative aspects of the study, the ERCs will only be notified.

Where data recorded in the eCRF and source document depart from the protocol, this will be reported as a protocol deviation or violation. These will be documented on a protocol deviations or violations log describing this departure, the circumstances requiring or resulting in it, and measures taken to avoid recurrence. All protocol deviations and violations will be reported to the ERCs.

## 9.2 Regulatory Notification

The study protocol and all requisite documents will be approved by the relevant regulatory authority in each country prior to study initiation:

- Kenya: The National Commission for Science, Technology and Innovation (NACOSTI)
- Tanzania: National Health Research Ethics Committee and the Muhimbili University of Health and Allied Sciences Research Ethics Committee
- Mozambique: Comité Nacional de Bioética para Saúde (CNBS)
- Lesotho: National Health Research Ethics Committee

## 9.3 Publication Policy

Following completion of the study, findings of the research are planned for oral presentation at scientific conferences and publication in peer reviewed scientific journals. The policy of the International Committee on Medical Journal Editors (ICMJE) member journals will be adhered to. Interim analysis during the follow-up period may be submitted for presentation or publication if deemed of scientific value by the Chief Investigator.

The Chief Investigator will formulate plans, organize data and prepare manuscripts for publication. Relationships with funding organizations, any other potential conflicts of interest, and the role of the funder will be disclosed in all manuscript submissions. Funding organizations will have the opportunity to review and comment on conference abstracts and manuscripts before submission but will not be involved in the decision to publish. All co-authors will have access to all data, and the Chief Investigator will have final decision on publication.

## 9.4 Sample Shipment Processing

All samples will be transported and processed following the standard operating procedures in the laboratory manual.

In Kenya, Tanzania and Mozambique all samples will be processed locally. Samples for DRT in Lesotho will be shipped to South Africa for drug resistance testing.

All samples sent to the laboratory will be de-identified; each sample will be allocated a research number. Patient names and other potentially identifying information will be removed. All samples will be destroyed once analysed according to international laboratory standards and GCLP guidelines. Samples will be used strictly for the purposes of the study and there will be no secondary data mining by the laboratory or other researchers.

# 10 References

1. The State of HIV Treatment, Testing, and Prevention in Low- and Middle-Income Countries; HIV Market Report 2023. Boston, MA, USA: Clinton Health Access Initiative, **2023**.

2. Consolidated Guidelines on HIV Prevention, Testing, Treatment, Service Delivery and Monitoring: Recommendations for a Public Health Approach. Geneva: World Health Organization, **2021**.

3. Kenya HIV Prevention and Treatment Guidelines. 2022 ed. Nairobi: National AIDS/STI Control Program, Ministry of Health Kenya, **2022**.

4. HIV care guide for adults, pregnant teenagers, lactating mothers and children: National STI, HIV and AIDS Control Program, National Directorate of Public Health, Ministry of Health Mozambique, **2023**.

5. National guidelines for the management of HIV and AIDS: National AIDS Control Programme, Ministry of Health, Community Development, Gender, Elderly and Children of Tanzania, **2019**.

6. Kingwara L, Onwonga V, Madada R, et al. Dolutegravir Resistance in Resource-Limited Settings. Conference on Retroviruses and Opportunistic Infections. Denver, USA, **2024**.

7. Chu C, Tao K, Kouamou V, et al. Prevalence of Emergent Dolutegravir Resistance Mutations in People Living with HIV: A Rapid Scoping Review. Viruses **2024**; 16(3): 399.

8. Bello G, Pals S, Bighignoli B, et al. Emerging Dolutegravir Resistance Among Children Being Investigated for Treatment Failure in Malawi. Conference on Retroviruses and Opportunistic Infections. Denver, USA, **2024**.

9. Ismael N. HIV drug resistance profile in clients experiencing treatment failure after the transition to dolutegravir-based first-line antiretroviral treatment in Mozambique. International Workshop on HIV Drug Resistance and Treatment Cape Town, South Africa, **2023**.

10. Bosch B, Sokhela S, Akpomiemie G, et al. High rates of long-term HIV RNA re-suppression after virological failure on dolutegravir in the ADVANCE trial. 12th IAS Conference on HIV Science. Brisbane, Australia, **2023**.

11. Tao K, Rhee SY, Chu C, et al. Treatment Emergent Dolutegravir Resistance Mutations in Individuals Naive to HIV-1 Integrase Inhibitors: A Rapid Scoping Review. Viruses **2023**; 15(9).

12. Akil B, Blick G, Hagins DP, et al. Dolutegravir versus placebo in subjects harbouring HIV-1 with integrase inhibitor resistance associated substitutions: 48-week results from VIKING-4, a randomized study. Antivir Ther **2015**; 20(3): 343-8.

13. Castagna A, Maggiolo F, Penco G, et al. Dolutegravir in antiretroviral-experienced patients with raltegravir- and/or elvitegravir-resistant HIV-1: 24-week results of the phase III VIKING-3 study. J Infect Dis **2014**; 210(3): 354-62.

14. Eron JJ, Clotet B, Durant J, et al. Safety and efficacy of dolutegravir in treatment-experienced subjects with raltegravir-resistant HIV type 1 infection: 24-week results of the VIKING Study. J Infect Dis **2013**; 207(5): 740-8.

15. Naeger LK, Harrington P, Komatsu T, Deming D. Effect of dolutegravir functional monotherapy on HIV-1 virological response in integrase strand transfer inhibitor resistant patients. Antivir Ther **2016**; 21(6): 481-8.

16. Paton NI, Musaazi J, Kityo C, et al. Dolutegravir or Darunavir in Combination with Zidovudine or Tenofovir to Treat HIV. N Engl J Med **2021**; 385(4): 330-41.

17. Kanise H, van Oosterhout JJ, Bisani P, et al. Virological Findings and Treatment Outcomes of Cases That Developed Dolutegravir Resistance in Malawi's National HIV Treatment Program. Viruses **2023**; 16(1).

18. Brown J, Ringera I, Luoga E, et al. GIVE MOVE: Randomized Trial on Genotype-Informed Management of Viremia in Children and Adolescents. Conference on Retroviruses and Opportunistic Infections. Denver, USA, **2024**.

# 11 Protocol Signature Page

I agree to conduct the trial in accordance with GCP and the applicable regulatory

requirements and with the approved protocol.

I agree to comply with the procedures for data recording / reporting.

I agree to permit monitoring, auditing and inspection and to retain the trial related

essential documentation for the period of time required according to ICH-GCP.

Name of Chief Investigator: Loice Achieng Ombajo

Signature:

Date:

Country Principal Investigators:

| Country | Name | Signature | Date |
| --- | --- | --- | --- |
| Mozambique | Nalia Ismael |  |  |
| Tanzania | Patricia Munseri |  |  |
| Lesotho | Irene Ayakaka |  |  |

# 12 Appendices

Appendix 1: Participating Study Sites

Appendix 2: Study Flowchart

Appendix 3: Study Timeline

Appendix 4: Participant Information Sheet and Consent Forms (ICFs)

Appendix 5: Case Report Forms (CRFs)

Appendix 6: Enhanced Adherence Counseling Protocols

Appendix 7: Research Team and Roles

Appendix 8: Investigators CVs and GCP

Appendix 9: Ndovu Steering Committee Charter

Appendix 10: Study budget

Appendix 11: Confirmation of Funding

Appendix 12: Letters of Support
