# Supplementary material for: The dolutegravir failure cohort: A multi-country longitudinal cohort with a randomised clinical trial of continued dolutegravir versus switch to darunavir in people with viraemia while on dolutegravir in Sub-Saharan Africa (The Ndovu Study) protocol
Source: PLoS One. 2026 Mar 13;21(3):e0330792. doi: 10.1371/journal.pone.0330792 (PMC12987441; doi:10.1371/journal.pone.0330792)
Supplement: S5 File — (DOCX) [file pone.0330792.s005.docx]

**Clinical Protocol**

**Investigating the optimal management of dolutegravir resistance: an open-label randomised controlled trial of maintaining dolutegravir or switch to ritonavir-boosted darunavir**

**Short Title: Ndovu RCT**

**ClinicalTrials.gov Identifier: NCT06747507**

**Sponsor: University of Nairobi**

This is a collaborative study between the University of Nairobi, acting as Sponsor, and Instituto Nacional de Saúde (INS), Muhimbili University of Health and Allied Sciences (MUHAS), SolidarMed, and the London School of Hygiene and Tropical Medicine

**Funding: Bill & Melinda Gates Foundation**

**Protocol Version 1.2: 24-February-2025**

# Investigators

**Chief Investigator**

Loice Achieng Ombajo, MBChB, M.Med, DLSHTM, MSc (ID), FRCP

**Principal Investigators**

Principal Investigator, Mozambique: Nalia Ismael, BSc, MSc, PhD

Principal Investigator, Tanzania: Patricia Munseri, MD, MMed, MPH, PhD

Principal Investigator, Lesotho: Irene Ayakaka, MBChB, MPH, MRes

**Co-Investigators**

Co-investigators – Kenya:

Jeremy Penner, MD, MHSc, DTM&H, CCFP, FCFP

Emily Wangui Kamau, MBChB, M.Med, MSc (ID)

Patrick Amoth, MBChB, M.Med

Andrew Mulwa, MBChB, Msc

Elizabeth Abong’o, KRCHN

Leonard Kingwara, BSc, MPH, PhD

Dalton C. Wamalwa, MBChB, M.Med, MPH

James Wagude, MBChB, M.Med

Rose Wafula, MBChB, MPH

Lazarus Momanyi, MBChB, MPH

Joseph Nkuranga, MBChB, MSc (Epi)

Florentius Ndinya, MBChB, M.Med,

Anne-Marie Macharia, MBChB, M.Med

Simon Wahome, BPharm, MPharm

Anthony Kiplagat, DCM, BA, MSc

Caroline Wafula, BPharm, MPharm, MBA

Lisa Abuogi, BA, MD, Res, MSc

Rena Patel, BA, M Phil, MD, MPH

Co-Investigators – Mozambique:

Raquel Matavele Chissumba, BSc, MSc, PHD

Patricia Maria Ramgi, MD

Co-Investigators – Tanzania:

Muhammad Bakari, MD, M.Med, PhD

Jamila Said Didi, MD, MBA, M.Med, MSc (Nephrology)

Co-Investigators – Lesotho:

Niklaus Labhardt, MD, MIH, FMH

Anna Klicpera, MD, DTM, MSc

Tapiwa Tarumbiswa MBBS, MBA

Co-Investigators – London School of Hygiene and Tropical Medicine (LSHTM):

Daniel James Grint, BSc, MSc, PhD

Charles Opondo, BPharm, MSc, PhD

# Protocol Amendment Summary of Changes Table

**Document History**

| Protocol Version | Date |
| --- | --- |
| 1.0 (Original version) | 04-October-2024 |
| 1.1 | 27-November-2024 |
| 1.2 | 24-February-2025 |

**Summary of Changes**

The changes made to version 1.1 of the protocol are summarized below:

| **Section and page number** | **Description of change** | **Brief rationale** |
| --- | --- | --- |
| Cover page and footers | Updated protocol version and date | To differentiate the versions of the protocols |
| Abstract, Page iv | Updated to include the target sample size, study sites and describe analysis methods | Enrich the abstract in-line with review comments |
| Secondary Objectives, Page 8 | Added objectives  “To evaluate the incidence of adverse events by study arm”  “To assess viral suppression based on prior PI or INSTI exposure”  “To assess viral suppression based on prior PI or INSTI failure” | To include an objective assessing safety using from the clinical and laboratory data that is collected  To include an objective that would include sub-analysis on viral suppression based on prior PI or INSTI exposure or failure-so as to include treatment experienced participants who are on 3^rd^ line regimens |
| Figure 1 Ndovu RCT Schema, Page 10 and Appendix 2 | Deleted “follow-up” in the screening section as a follow-up VL is not part of the inclusion criteria | Edits made to the schema to align with other sections of the protocol |
| Time and Events Table, Pages 20 and 21 | Footnote added to specify that height will be measured during each visit for participants < 18 years old | To clarify that height for adults will be measured once to align with other study tools and standard clinical practice |
| Assessment at Each Visit, Pages 23 | Specified MUAC will be measured for children aged 3 to 5 years old; and corrected the cuff size for blood pressure measurement | Corrections and additional clarifications to guide conduct of the study |
| Methodology  6.3 Blood Sampling  Page 25  6.3.1 Sample Processing in Kenya | Edited total volume of blood which will be collected from each study participant to 70ml from 65 ml  Added additional information on how different machines will yield consistent results across the board despite the machine being used so as to ensure there will be no differences or deviations in the results. This is | To rectify initial error made in calculation of total blood sample which will be collected  This was in response to JOOTRH ISERC comments |
| Data Management  Section 9.4 | Added additional details on storage duration and data destruction methods | In line with JOOTRH ISERC comments |
| Site Specific Addendum for Kenya | Included RCT Site Specific addendum including roles of other countries and specifically Kenya in addressing recruitment, RCT sites, local regimens to be used, and data protection considerations | In line with ERC review comments |
| Participant information sheets and consent forms, Appendix 5 | Grammatic edits and correction of typographic errors | For clarity and to aid understanding by participants |
|  | Labelling of each participant information sheet and consent form detailing who it is for and at what age it should be used  Indicated age of assent clearly to be 12-17 years for those who have been disclosed to their HIV status  Updated the total amount of blood to be drawn doe the study to 70ml from 65ml | To avoid wrong application of forms to participants and align to the protocol  To rectify error made in the actual calculation-it should be 70ml and not 65ml |
|  | Section 17 of appendices 5A, 5B and 5C: Included information concerning sample shipment for processing in South Africa for TFV-DP levels and storage of samples collected for DRT  Included consent for sample storage and sample shipment and processing in South Africa | In line with ERC review comments |
| Case Report Forms, Appendix 6 | Page 28: Included head circumference | In line with protocol inclusion of head circumference as an anthropometric measurement for children |
|  | Screening CRF: Included specific DTG DRMs that are part of inclusion criteria in protocol | In line with protocol |
|  | Screening CRF: Added VL if test result is older than 3 months | In line with protocol |
|  | Screening CRF: Included regimens ABC/3TC/NVP and AZT/3TC/LPVr as prior regimens  Included dosage and frequency | To avoid these as ‘other’ options in analysis  For paediatric regimens dosed according to weight |
|  | EAC form: Included clinical/medical causes of impaired adherence-poor absorption, inadequate dosing (paediatrics), included options for barriers assessed and plans to mitigate them in individualized treatment plan check list and additional barriers | For wholistic assessment of poor adherence |
|  | Treatment Satisfaction (HIVTSQ): Included HIVTSQc | In line with protocol, to be done at 6 months to reflect if there is change in HIV treatment satisfaction |
|  | Follow-up CRF: Included provision for study exit if there is substitution of DTG or DRV | In line with protocol |
|  | Follow-up CRF: Included whether pregnancy was done and results of pregnancy test into follow up CRF, removing it from lab section | It will be done point-of-care so the result will be available before the visit is over. It will be easier to include it in the CRF instead of entering in a separate lab result for like for labs whose results come later |
|  | HIV DRT Form: NRTI resistance: include “Resistance to abacavir, Zidovudine and  14: for first “I” included as it was missing in "INSTI" | To align with protocol |
|  | Lab HIV RNA CRF: After number 7 in italic corrected to VL ≥ 200 copies/mL (instead of 400) | To align with protocol |
|  | Study Conclusion CRF: Edited as follows  • Reasons for conclusion, break first bullet point into three points and edit to read as follows  o Early failure based on HIV-1 RNA viral load ≥ 0.3 log from most recent prior value at month 1  o Early failure based on HIV-1 RNA viral load ≥ 0.3 log from most recent prior value at month 3  o Early failure based on new major DTG- or DRV/r-associated DRM  • Removed “Confirmation of” from all reasons (we are not repeating VL to confirm results) | To align with protocol |

# Abstract

**Background**

The majority of people living with HIV (PLWH) on first line antiretroviral therapy (ART) in low- and middle-income countries are on dolutegravir (DTG)-containing regimens. Different countries have adopted different approaches in the management of people on DTG-based first line ART with repeat HIV viral load (VL) of > 1,000 copies/mL after 3 months of enhanced adherence counseling. For example, Kenya recommends a drug resistance test (DRT) to guide on switch and the optimal second-line regimen; Mozambique and Tanzania recommend switch to 2 nucleoside reverse transcriptase inhibitors (NRTIs) and protease inhibitors (PIs) without drug resistance testing; South Africa does not recommend switch from DTG or DRT for those who are on first-line DTG-containing regimens within the first 2 years of treatment, after which management is guided by possible DRT and expert opinion. The World Health Organization has recognised the role of drug resistance testing (DRT) in a treatment failure algorithm for people living with HIV receiving DTG-based treatment to minimise unnecessary switches from this regimen. The switch to PI has disadvantages including higher cost, higher pill burden, less convenient administration (often should be taken with food), more potential drug-drug interactions, poorer tolerability and more long-term toxicities.

**Goal**

To assess the efficacy and safety of remaining on DTG compared to switching to DRV/r among people failing DTG-based ART with at least one major DTG drug resistance mutation (DRM).

**Methods**

This is a phase 3b, multi-country, open label, two arm, active controlled randomized clinical trial (RCT) over 12 months describing the efficacy and safety of switching from DTG to DRV/r among PLWH age ≥ 3 years who are failing DTG-based ART with HIV-1 RNA ≥ 200 copies/mL and ≥ 1 major DTG-associated DRM (and most recent prior HIV-1 RNA ≥ 1,000 copies/mL after at least 6 months on DTG-based ART). The primary efficacy endpoint is the proportion of participants with HIV-1 RNA < 200 copies/mL at month 6. The study will be conducted in 9 sites in Kenya, Mozambique, Tanzania and Lesotho targeting 392 participants including 30 children aged between 3 and 14 years old. The primary efficacy analysis will assess the difference in proportion of participants with viral suppression at month 6 using Cochran-Mantel-Haenszel method. This RCT is nested within an observational cohort study describing HIV-1 viral suppression of people with HIV-1 RNA value of ≥ 1,000 copies/mL after at least six months on DTG-based ART (Ndovu Cohort Study, submitted as a separate protocol).

**Study Utility**

This RCT will address the gap in published data on the effect of DTG-associated DRMs on viral suppression among people remaining on DTG-based ART while using current NRTI options, as well as the gap on the optimal management strategy for this population.

# Abbreviations/Acronyms

| 3TC | Lamivudine |
| --- | --- |
| AE | Adverse event |
| ALT | Alanine transaminase |
| ART | Antiretroviral therapy |
| AST | Aspartate transaminase |
| AZT | Azidothymidine or zidovudine |
| CCC | Comprehensive care clinic |
| CD4 | Clusters of differentiation 4 |
| CNBS | Comité Nacional de Bioética para Saúde |
| COVID-19 | Coronavirus disease |
| Cr | Creatinine |
| CRF | Case report form |
| DAIDS | Division of AIDS |
| DBS | Dried blood spot |
| DNA | Deoxyribonucleic acid |
| DRM | Drug resistance mutation |
| DRT | Drug resistance test |
| DRV/r | Ritonavir boosted darunavir |
| DSMB | Data safety monitoring board |
| DTG | Dolutegravir |
| eCRF | Electronic case reporting form |
| EFV | Efavirenz |
| ERC | Ethics Review Committee |
| FDA | Federal Drug Authority |
| FTC | Emtricitabine |
| GCP | Good clinical practice |
| HIV | Human Immunodeficiency Virus |
| HIVTSQ | HIV treatment satisfaction questionnaire |
| HIVTSQc | HIV treatment satisfaction questionnaire change version |
| HIVTSQs | HIV treatment satisfaction questionnaire status version |
| INS | Instituto Nacional de Saúde |
| INSTI | Integrase strand transfer inhibitor |
| IP | Investigational product |
| ISERC | Institutional Scientific and Ethics Review Committee |
| JOOTRH | Jaramogi Oginga Odinga Teaching and Referral Hospital |
| KNH | Kenyatta National Hospital |
| LDL | Low-density lipoprotein |
| MUHAS | Muhimbili University of Health and Allied Sciences |
| NNRTI | Non-nucleoside reverse transcriptase inhibitor |
| NRTI | Nucleoside reverse transcriptase inhibitor |
| NVP | Nevirapine |
| PEPFAR | Presidential emergency plan for AIDS relief |
| PI | Protease inhibitor |
| PLWH | People living with HIV |
| RCT | Randomized clinical trial |
| RNA | Ribonucleic acid |
| SAE | Serious adverse event |
| TAF | Tenofovir alafenamide |
| TDF | Tenofovir disoproxil fumarate |
| TFV-DP | Tenofovir-diphosphate |
| ULN | Upper limit of normal |
| UoN | University of Nairobi |
| VL | Viral load |
| WHO | World Health Organization |

**Investigating the optimal management of dolutegravir resistance: an open-label randomised controlled trial of maintaining dolutegravir or switch to ritonavir-boosted darunavir**

**Table of Contents**

Investigators ii

Protocol Amendment Summary of Changes Table iii

Abstract vii

Abbreviations/Acronyms viii

1. Introduction 3

1.1 Background 3

1.2 Literature Review 3

1.3 Justification 6

1.4 Hypothesis 6

2. Specific Aims 7

2.1 Goal 7

2.2 Specific Objectives 7

2.3 Endpoints 8

3. Study Design 9

3.1 Overview of Study Design 9

4.1 Number of Participants and Participant Selection 11

4.2 Recruitment 11

4.3 Inclusion Criteria 11

4.4 Exclusion Criteria 12

4.5 Duration of Involvement 12

4.6 Withdrawal of Participants and Discontinuation Criteria 12

4.7 Loss to Follow-up 13

4.8 The End of the Study 13

5 Treatment of Participants 14

5.1 Study Treatment Regimens 14

5.2 Study Drug Information 14

5.3 Prior and Concomitant Therapy 17

5.4 Treatment Compliance 17

5.5 Provision of Treatment After the End of the Study 18

6 Study Assessments and Procedures 19

6.1 Time and Events Schedule 19

6.2 Assessments at Each Visit 20

6.3 Blood Sampling 22

6.4 Qualitative Interviews 24

6.5 Clinical Evaluation 26

6.6 Samples for Future Research 26

7 Statistical Methods 27

7.1 Analysis Populations 27

7.2 Efficacy Analysis 27

7.3 Safety Analysis 28

7.4 Qualitative Analysis 28

7.5 Interim Analysis 29

7.6 Software Used 29

8. Adverse Events 30

8.1 Definition of an Adverse Event (AE) 30

8.2 Definition of a Serious Adverse Event (SAE) 30

8.3 Assessment of Severity 31

8.4 Assessment of Causality 31

8.5 Collection and Follow-up of AEs 31

8.6 Methods of Detecting AEs and SAEs 32

9 Data Handling 33

9.1 Recording of Data 33

9.2 Source Documentation and Study Records 33

9.3 Data Management 33

9.4 Storage of Data 34

10 Quality Control and Quality Assurance 35

10.1 Monitoring Arrangements 35

10.2 Quality Assurance 35

11 Ethical Considerations 36

11.1 Ethical Approvals 36

11.2 Informed Consent 36

11.3 Study-specific Design Considerations 37

11.4 Clinical Trial Insurance 38

11.5 Participant Compensation 38

11.6 Privacy of Personal Data 38

11.7 Independent Data Safety Monitoring Board 39

12. Administrative Procedures 41

12.1 Protocol Modifications 41

12.2 Regulatory Notification 41

12.3 Publication Policy 42

12.4 Drug Accountability 42

12.5 Sample Shipment Processing 42

13 References 43

14 Protocol Signature Page 46

15 Appendices 47

# 1. Introduction

## 1.1 Background

The majority of people living with HIV (PLWH) on first line antiretroviral therapy (ART) in low- and middle-income countries are on dolutegravir (DTG)-containing regimens [1]. Current World Health Organization (WHO) guidelines recommend that people on DTG-based first line ART with HIV viral load > 1,000 copies/mL should undergo enhanced adherence counselling and, if viral load remains > 1,000 copies/mL after 3 months, they should be switched to a protease inhibitor (PI)-based second line regimen [2]. Different countries have adopted different approaches in their guidelines. For example, Kenya recommends that after intensified adherence counselling, patients with persistent viraemia > 1,000 copies/mL should receive a drug resistance test (DRT) to guide switch and the choice of the optimal second-line regimen [3]; Mozambique, Tanzania and Lesotho recommend switch to 2 nucleoside reverse transcriptase inhibitors (NRTIs) and PI after failure of a DTG-containing first-line regimen [4, 5]; South Africa does not recommend switch from DTG or DRT for those who are on first-line DTG-containing regimens within the first 2 years of treatment, after which management is guided by possible DRT and expert opinion [6]. The switch to PI has disadvantages including higher cost, higher pill burden, less convenient administration (often should be taken with food), more potential drug-drug interactions, poorer tolerability and more long-term toxicities.

## 1.2 Literature Review

The WHO recommendation to switch to PI-based therapy is based on the untested assumptions that people failing DTG have selected for clinically relevant integrase inhibitor drug resistance mutations (DRMs) and are therefore more likely to achieve viral suppression with a change in regimen compared to remaining on a DTG-containing regimen. Clinical trial and cohort data show that DTG-based ART rarely leads to virological failure, and that the prevalence of emergent DTG-associated DRMs is low among those with failure [7]. Cross-sectional data show varying rates of DTG-associated DRMs among DRTs performed; however, the frequent lack of reporting of denominators on total number of people receiving DTG or total number of people with virologic failure limit the conclusions we can make about the prevalence of DTG-associated DRMs among people with virological failure [7]. Despite low proportions of people with virologic failure and emergent DTG-associated DRMs, the absolute number of people failing DTG-based regimens has important implications for ART programs.

The WHO has recognised the role of drug resistance testing (DRT) in a treatment failure algorithm for people living with HIV receiving DTG-based treatment to minimise unnecessary switches from this regimen [2]. However, routine DRT is unlikely to be available in many resource limited settings in the near future. The GIVE MOVE trial, in Lesotho and Tanzania, randomised children and adolescents with recent viraemia on first-line ART to the usual care arm (which consisted of a viral load-informed treatment) or to a DRT arm in which DRT and expert review informed care; they found no significant difference in the primary outcome (death, hospitalisation, new WHO stage 4 event or VL ≥50 copies/ml) between the two groups [8].

Emerging data from African countries have shown varying levels of integrase drug resistance mutations. Results from a recent cross-sectional survey in Malawi in children on DTG with confirmed virologic failure, found major INSTI DRM in 16.3% of 133 samples that were successfully sequenced [9], while in Mozambique, DTG resistance was found in 19.6% of 183 samples from patients with virologic failure [10]. In Kenya, surveillance samples from patients with viral non-suppression show a prevalence of up to 22.6% in ART experienced patients (on DTG as second or third line regimens) and 8.3% in those on failing a first line DTG regimen [11].

Most people failing first-line DTG-based regimens without DTG-associated DRMs can be expected to re-suppress without a change in regimen if adherence and potential drug interactions are addressed, and therefore would not benefit from a change to PI-based regimen [12]. For PLWH who are failing DTG-based regimens and have developed DTG-associated DRMs, there is very limited direct evidence to guide their management. Data on pathways of DTG-associated DRMs and their effects on in-vitro DTG susceptibility is accumulating [13], with substitutions at eight codons currently known to contribute to reduced DTG susceptibility, and thus considered “major” DTG-associated DRMs: 66K, 92Q, 118R, 138K/A/T, 140S/A/C, 148H/R/K, 155H and 263K (Stanford HIV Drug Resistance Database version 9.6, last updated 9-Mar-2024). How the genotypic resistance patterns relate to the in-vivo virologic response to a DTG-containing ART regimen is uncertain, and multiple management strategies are currently being used including: increasing DTG to twice-daily dosing, switching from DTG to a PI, adding a PI to the DTG-based regimen, among others.

In a recent scoping review, Tao and colleagues found that major INSTI-associated DRMs clustered into four signature positions including R263K, G118R, N155H and Q148H with minimal overlap [13]. The majority of viruses had just one signature mutation, predominantly R263K. Other than G118R, the other DRMs alone were not associated with high levels of reduced DTG susceptibility and studies to determine the significance of these on clinical management are needed.

The VIKING trials assessed the efficacy of twice daily DTG plus an optimised background regimen in heavily treatment experience patients who had previously received a first generation INSTI and had INSTI DRMs [14-17]. VIKING was a single-arm phase IIb study that initially evaluated once-daily DTG; however, poor virologic response in the initial cohort prompted a protocol change to test twice-daily DTG. Viral suppression to <50 copies/ml at week 24 was achieved by 41% of participants in the once-daily DTG cohort, and by 75% in the twice-daily DTG cohort [16]. VIKING-3 was a single-arm phase III study which further evaluated the DTG twice-daily plus optimized background therapy strategy, with 69% achieving viral suppression to <50 copies/ml by week 24 [15]. Extrapolating the VIKING series of results to the population of patients failing a first line DTG-based regimen with DTG-associated DRMs has limitations: the VIKING participants were highly treatment experienced which may be an indicator of more extreme adherence challenges; they had resistance to at least 3 drug classes which may have synergistic effects on virologic response, and recognized INSTI DRMs at the time were different than current standards. Given that some participants in VIKING achieved viral suppression on single-dose DTG despite INSTI DRMs, it is reasonable to test whether single-dose DTG could provide a better response in a population that is less extensively treatment experienced.

Evidence for switching from DTG to a PI is extrapolated from studies where participants failed a non-DTG first-line regimen and were treated with a PI-based second line regimen. Most relevant for our proposed study population is the NADIA trial which enrolled people failing an NNRTI plus Tenofovir disoproxyl fumarate (TDF) / Lamivudine (3TC) and randomized them to second line therapy of either DTG or DRV/r, plus either TDF/3TC or AZT/3TC [18]. At week 48, 92% of participants on DRV/r achieved viral suppression <400 copies/ml compared to 90.2% on DTG, a non-statistically significant difference between the arms. A limitation of extrapolating these results (and those of other second-line PI studies) to the population of patients failing a first line DTG-based regimen with DTG-associated DRMs is that DTG-based first-line is simpler to take and better tolerated than EFV or NVP, with a higher barrier to resistance, so people failing the DTG-based regimen may be a sub-group with more barriers to adherence.

Real-world suppression rates of patients with documented DTG-associated DRMs have been published from national program data in Malawi [19]. Among the 24 patients with DTG resistance, 18 had been on two or more regimens prior to DTG. Of 11 patients with follow-up viral load data available after implementing DRT-based regimen changes (either 2NRTI + PI/r; 2NRTI + PI/r + DTG; 2NRTI + PI/r + DTG + DTG; 2 NRTI + DTG + DTG), 9 (82%) achieved a viral load < 200 copies/mL. All participants who received double-dose DTG (4/4) achieved viral suppression, and 5/7 who received PI/r or PI/r + single-dose DTG achieved viral suppression. Of two patients who remained on DTG without a change in regimen despite DTG-associated DRMs, 1 of them re-suppressed.

For selection of NRTIs among people switching regimens due to treatment failure, WHO recommends changing the NRTI based on predicted or assumed DRMs and the genotypic resistance patterns associated with these DRMs, switching from TDF or abacavir (ABC) in first line to zidovudine (AZT) in second line, and from AZT in first line to TDF in second line [2]. Multiple studies have demonstrated that NRTIs still contribute to viral suppression when combined with lopinavir/r, even when the documented DRMs predict no NRTI activity in the regimen [20-24]. Similarly, the NADIA trial has demonstrated that among people failing a TDF-containing regimen, those who remained on TDF in second line (combined with either DTG or DRV/r) had superior virologic outcomes at 96 weeks compared to those who switched to AZT in second line (92% suppression on TDF compared to 85% on AZT, superiority p=0.019) [25]. TDF has several advantages over AZT including superior efficacy in first- and second-line, once-daily dosing, and fewer treatment-limiting adverse events [25-27].

To understand the efficacy of remaining on DTG-containing ART despite having virologic failure, and particularly amongst those with DTG-associated DRMs, we will need to be able to assess outcomes based on adherence levels. Viral suppression while remaining on DTG in the presence of DRMs may depend on a threshold level of adherence, and this threshold could vary with the DRM pattern. Pharmacokinetic studies have found that intracellular tenofovir-diphosphate (TFV-DP) levels in dried blood spots (DBS) correlate with cumulative TDF drug exposure in the preceding 6-8 weeks, with threshold TFV-DP levels defined for TDF adherence levels [28, 29]. TFV-DP concentrations have also shown strong association with virologic suppression for study participants on TDF [30-32] and TAF [33], as well as association with directly measured adherence on TDF [34] and TAF [35]. Among people suppressed on TDF-containing ART, TFV-DP levels in DBS also predict future viremia [32, 36-38] and development of DRMs [38].

## 1.3 Justification

Taken together, a range of information using different viral load suppression thresholds, different approaches to DRT, and unproven extrapolation from other studies currently inform our understanding of DTG DR management. While we do not expect widespread, routine program access to DRT or TFV-DP levels, there is an opportunity to conduct a special study that, in the context of enhanced adherence monitoring and support, will provide the scientific underpinning for whether and when viremia with DTG resistance requires regimen switch – and in turn inform whether and when DRT is needed when viremia with DTG is detected through routine viral load screening. Additionally, very limited data exists for DTG-associated DRMs and their clinical significance among children.

We seek to address the gap in published data on the effect of DTG-associated DRMs on viral suppression among people remaining on DTG-based ART while using current NRTI options, as well as the gap on the optimal management strategy for this population. We will evaluate the efficacy and safety of remaining on DTG compared to switching to DRV/r in a randomized clinical trial over 12 months (the Ndovu RCT). We will also evaluate the viral suppression and emergence of DRMs among PLWH with sustained viremia on DTG-based ART through a prospective cohort evaluation, which will identify potential participants for the RCT (the Ndovu Cohort Study, submitted as a separate study protocol).

The data generated from these studies will inform WHO and national guidelines on management of DTG failure.

## 1.4 Hypothesis

Among people with confirmed virologic failure on DTG-based ART with at least one documented major DTG-associated DRM, switching to DRV/r is no different to remaining on DTG, as measured by HIV-1 RNA < 200 copies/mL within 6 months from randomization.

# 2. Specific Aims

## 2.1 Goal

- To assess the efficacy and safety of remaining on DTG compared to switching to DRV/r among people failing DTG-based ART with at least one major DTG DRM

## 2.2 Specific Objectives

- **Primary objective**
  - To assess the comparative efficacy of switching to DRV/r-based regimen after confirmed virologic failure and of remaining on DTG-based ART in achieving viral suppression of <200 copies/mL at 6 months from randomization among participants with ≥1 major DTG-associated DRM
- **Secondary objectives**
  - To evaluate difference in viral suppression to HIV-RNA < 200 copies/mL at 12 months from randomization
  - To evaluate if switching to DRV/r-based ART after virologic failure is superior to remaining on DTG-based ART in achieving viral suppression to <200 copies/mL at 6 months from randomisation
  - To evaluate difference in viral suppression using HIV-RNA cut-off of < 50 copies/mL at 6 and 12 months from randomization
  - To evaluate difference in viral suppression using HIV-RNA cut-off of < 1,000 copies/mL at 6 and 12 months from randomization
  - To assess viral suppression among different age strata (3-9 years, 10-19 years, ≥20 years, 20-24 years, 25-34 years, 35-44 years, ≥45 years)
  - To assess viral suppression based on prior PI or INSTI exposure
  - To assess viral suppression based on prior PI or INSTI failure
  - To assess viral suppression based on participant sex
  - To evaluate the incidence of adverse events by study arm
  - To determine adherence levels, based on DBS TFV-DP levels, associated with: suppression; selection of treatment-emergent DRMs
  - To determine incidence of treatment-emergent DRMs
  - To describe patterns of accumulated DRMs
  - To evaluate DRM patterns associated with sustained non-suppression
  - To investigate predictors of development of DTG-associated DRMs (age, other non-INSTI DRMs, VL at switch to DTG, time with viremia, etc.)
  - To assess viral suppression based on pre-enrollment NRTI
  - To assess viral suppression based on TDF vs TAF as the study NRTI
  - To assess the impact of regimen on change in CD4 count
  - To assess the impact of regimen on participant satisfaction
  - To explore the experiences and acceptability of remaining on DTG containing ART or switching to DRV-containing ART for management of treatment failure to DTG based ART regimens from perspectives of health care providers, policy makers and PLWH enrolled in Ndovu study
  - To understand barriers and facilitators of managing treatment failure to DTG based regimens by:
    - remaining on DTG based regimens
    - switching to DRV based regimens among health care providers, policy makers and PLWH enrolled in Ndovu study

## 2.3 Endpoints

- **Primary endpoint**
  - Proportion of participants with HIV-1 RNA < 200 copies/mL at month 6 from randomization using a modified US Food and Drug Administration (FDA) snapshot algorithm [39].
- **Secondary endpoints**
  - Proportion of participants with viral suppression to HIV-RNA < 200 copies/mL at 12 months from randomization
  - Proportion of participants with viral suppression using HIV-RNA cut-off of < 50 copies/mL at 6 and 12 months from randomization
  - Proportion of participants with viral suppression using HIV-RNA cut-off of < 1,000 copies/mL at 6 and 12 months from randomization
  - Proportion of participants with viral suppression among different age strata (3-9 years, 10-14 years, 15-19 years, 20-24 years, 3-15 years, ≥15 years, 16-24 years, 25-34 years, 35-44 years, ≥45 years)
  - Proportion of participants with viral suppression based on prior PI or INSTI exposure
  - Proportion of participants with viral suppression based on prior confirmed or suspected virological failure while on PI or INSTI
  - Proportion of participants with viral suppression based on participant sex
  - Proportion of participants with viral suppression based on adherence levels, as measured by DBS TFV-DP levels
  - Proportion of participants with treatment-emergent DRMs based on adherence levels, as measured by DBS TFV-DP levels
  - Incidence of treatment-emergent DRMs
  - Genotypic resistance mutations
  - Proportion of participants with treatment-emergent DRMs based on age, other non-INSTI DRMs, VL at switch to DTG, time with viremia, and other factors
  - Proportion of participants with viral suppression based on pre-enrollment NRTI
  - Proportion of participants with viral suppression based on TDF vs TAF as the study NRTI
  - Change in CD4 count from baseline to month 6 and 12
  - Participant satisfaction (HIVTSQ) at baseline and month 6
  - Participant and provider experiences from qualitative interviews

# 3. Study Design

# 3.1 Overview of Study Design

This is a phase 3b, multi-country, open label, two arm, active controlled randomized clinical trial over 12 months describing the efficacy and safety of switching from DTG to DRV/r among PLWH age ≥ 3 years who are failing DTG-based ART with HIV-1 RNA ≥ 200 copies/mL and ≥ 1 major DTG-associated DRM (substitution at codon 66K, 92Q, 118R, 138K/A/T, 140S/A/C, 148H/R/K, 155H or 263K ), and most recent prior HIV-1 RNA ≥ 1,000 copies/mL after at least 6 months on DTG-based ART. The primary efficacy endpoint is the proportion of participants with HIV-1 RNA < 200 copies/mL at month 6. This RCT is nested within an observational cohort study describing HIV-1 viral suppression of people with HIV-1 RNA value of ≥ 1,000 copies/mL after at least six months on DTG-based ART (Ndovu Cohort Study, submitted as a separate protocol).

**Figure 1: Ndovu RCT Schema**

The study will enroll participants from the Ndovu cohort study and take place in nine sites in the four participating countries as outlined in table 1.

**Table 1: RCT Sites and Enrollment Targets**

| Country | **RCT Sites** | Proposed RCT enrolment target |
| --- | --- | --- |
| Kenya | - - Kenyatta National Hospital (KNH) in Nairobi County   - Jaramogi Oginga Odinga Teaching and Referral Hospital (JOOTRH) in Kisumu County   - Bomu Hospital Changamwe, in Mombasa County | 144 (132 adults + 12 children) |
| Tanzania | - - Muhimbili University of Health and Allied Sciences (MUHAS) Clinical Trial Unit in Dar es Salaam | 56 (50 adults + 6 children) |
| Mozambique | - - CS Machava II in Maputo Province   - CS Ndlavela in Maputo Province   - CS Ponta Gea in Sofala Province | 144 (132 adults + 12 children) |
| Lesotho | - - Butha-Buthe District Hospital in Butha Buthe District   - Mokhotlong Hospital in Mokhotlong District | 48 |
| **Total** | | **392** |

Where a site fails to meet the target numbers, other sites may enroll additional numbers.

These RCT sites currently have approximately 6,600 patients on DTG-based ART with VL ≥ 1,000 copies/mL (Appendix 1).

Study visits will take place at screening/enrolment and months 1, 3, 6, 9 and 12. HIV-1 RNA viral load will be performed at months 1, 3, 6, 9 and 12. If HIV-1 RNA is ≥ 200 copies/mL at month 3 and 6, then genotypic drug resistance testing will be performed. DBS for TFV-DP levels will be taken at months 1 and 6. Other study investigations include CD4 count, complete blood count, serum Cr, ALT, AST and patient satisfaction questionnaires (HIVTSQ). The schedule of investigations is outlined in the Time and Events table (section 6.1).

**3.2 Randomization and Blinding**

Participants will be randomized at baseline in a 1:1 ratio using a computer-generated randomization sequence to either remain on a DTG-based regimen or switch to a DRV/r-based regimen. Randomization will be stratified to ensure 362 participants are ≥ 15 years old, and 30 participants are between age 3 and 14 years old.

**4 Study Population**

## 4.1 Number of Participants and Participant Selection

The randomised controlled trial will test the hypothesis that switching to DRV/r-based therapy is no different to remaining on DTG. The primary outcome of the trial is viral suppression 6 months after randomisation, using a modified FDA snapshot algorithm using HIV-RNA VL cut-off of < 200 copies/mL.

The sample size of the trial has been calculated based on the following assumptions:

- 5.5% of participants enrolled in the cohort who fail to suppress HIV-VL with enhanced adherence counselling will have ≥ 1 major DTG-associated DRM
- 55% of participants will achieve viral suppression on DTG
- 70% of participants will achieve viral suppression on DRV/r

The trial should have 80% power and a 5% type-I error rate while allowing for 10% attrition of the study population prior to the 6 month primary endpoint.

Research question: What is the most appropriate management strategy of PLWH with sustained virologic failure on DTG and DTG DRMs?

Required sample size N=362 (330 with additional 32 to account for a 10% loss to follow up), with 181 per arm. The aim is to be fully powered for the population aged above 15 years; since we expect 92% of Ndovu cohort participants to be ≥ 15 years. The target sample size of 181 participants per arm is sufficient to estimate the hypothesized suppression rates with a precision of the 95% confidence interval of approximately +/- 7%. In addition, we will enroll 30 children aged 3 to 14 years as a pediatric pilot (pediatric sample size determined using the proportion of children we expect enrolled in the cohort study), bring the total sample size to 392 participants. The sample size is computed using the artbin package in STATA statistical software. Full details on the formulation of the sample size formula are available in The Stata Journal [40].

## 4.2 Recruitment

Participants will be recruited without advertisement from the pool of participants in the Ndovu cohort study. The Ndovu cohort study is described in a separate protocol; in brief, the cohort is enrolling participants on DTG-based ART who are age ≥ 1 year, with most recent HIV-1 RNA ≥ 1,000 copies/mL within 3 months prior to enrollment and taken after at least 6 months on ART, and not currently on concomitant PI or NNRTI.

Ndovu cohort participants with ≥ 1 major DTG-associated DRM (whether from historic pre-enrolment DRT or DRT performed during the Ndovu cohort study) will be invited for screening into the Ndovu RCT at one of the clinical trial sites. Potential RCT participants who provide informed consent at screening will be assessed for eligibility.

## 4.3 Inclusion Criteria

Participants must satisfy all of the following criteria to be enrolled in the study:

- - - Enrolled in the Ndovu cohort study
    - Able and willing to understand and comply with the protocol requirements, instructions and restrictions
    - Able and willing to provide informed consent for the nested clinical trial (assent as appropriate and legal guardian consent if < 18 years)
    - Age ≥ 3 years
    - Most recent HIV-1 RNA ≥ 200 copies/mL
    - At least one major DTG-associated DRM (substitution at codon 66K, 92Q, 118R, 138K/A/T, 140S/A/C, 148H/R/K, 155H or 263K)

## 4.4 Exclusion Criteria

Potential participants who meet any of the following criteria will be excluded from participating in the study:

- Pregnant or breastfeeding
- Using any concomitant therapy disallowed as per the reference safety information and product labeling for the study drugs
- WHO stage 3 or 4 opportunistic infection which would prevent randomisation to either arm (e.g. due to drug interactions or significant liver or renal injury) within 4 weeks prior to RCT screening
- Investigator opinion that the potential participant should discontinue DTG immediately for clinical reasons
- Investigator opinion that the potential participant should not switch to DRV/r for clinical reasons

## 4.5 Duration of Involvement

Participants will be engaged in study for 12 months.

## 4.6 Withdrawal of Participants and Discontinuation Criteria

A participant is free to withdraw from the study at any time. In addition, the investigators may decide, for reasons of medical prudence, to stop study medications.

All participants who discontinue study medication will be followed up and requested to attend study visits up until month 12.

If any participant has an increase in HIV-1 RNA of ≥ 0.3 log from most recent prior value at month 1 or 3, or has any treatment-emergent major DTG-associated or DRV/r-associated DRM at month 3 (and adherent to ART), they will be classified as an early failure and be withdrawn from the RCT; management will be determined by the protocol-defined management algorithm and they will continue follow-up until month 12.

Study medication may also be discontinued in the following instances:

- If the participant withdraws their consent
- If the participant requires a substitution or discontinuation of DTG or DRV/r (e.g. for drug-drug interactions or toxicity)
- If the investigators consider in the interest of the participant (i.e. intercurrent illness, unacceptable toxicity) that it is best for them to stop study medication
- If the participant fails to comply with the protocol requirements or fails to cooperate with investigators

The date and reasons for the withdrawal will be clearly stated on the participant’s Case Report Form (CRF) and source document. Participants will be asked to continue attending all study visits.

## 4.7 Loss to Follow-up

A participant will be considered lost to follow-up if the participant repeatedly fails to return for scheduled visits and is unable to be contacted by the study site. The following actions will be taken if a participant fails to return to the clinic for a required study visit:

- The study team will attempt to contact the participant and reschedule the missed visit as soon as possible, counsel the participant on the importance of maintaining the assigned visit schedule and ascertain whether the participant wishes to and/or should continue in the study. Each participant who fails to return for a study visit should be discussed with the study coordinator/physician
- Before a participant is deemed lost to follow-up, every effort will be made to regain contact with the participant (where possible, 3 telephone calls, a home visit, and a text message)
- Contact attempts will be documented in the participant’s medical record
- Should the participant continue to be unreachable, the participant will be considered to have withdrawn from the study

## 4.8 The End of the Study

The end of the study is defined as the date of the last visit of the last participant undergoing follow up.

# 5 Treatment of Participants

# 5.1 Study Treatment Regimens

All participants who are randomized to remain on DTG will continue DTG-based ART for the duration of the study, unless they meet criteria for early failure in which case management is determined by the protocol-defined algorithm. All participants in the clinical trial who are randomized to switch to DRV/r will be switched to DRV/r and continue DRV/r for the duration of the study, unless they meet criteria for early failure in which case management is determined by the protocol-defined algorithm.

All participants in both study arms will receive TAF or TDF plus 3TC or FTC as the NRTI, depending on national guidelines and NRTI availability, unless there is a clinical reason not to use TDF or TAF. Switching NRTIs for clinical reasons during study follow up is allowed and does not result in discontinuation.

All ARVs will be prescribed following the age- and weight-based dosing and administration specifications in the product monographs for each ARV, including recommendations on administration with regard to food.

Participants will be dispensed a 4-week supply of ARVs at baseline, a 2-month supply at month 1, and a 3-month supply at months 3, 6 and 9.

# 5.2 Study Drug Information

Physical description:

DRV/r will be administered either as a fixed-dose combination of DRV and RTV 400mg/50mg, or as separate tablets of DRV 600mg and ritonavir 100mg.

DTG will be administered as a fixed-dose combination of DTG/3TC/TDF (beige, biconvex, oval or white biconvex oval)

Dosing:

- DTG/TDF/3TC: Given as a single tablet containing 50mg DTG/300mg TDF/300mg 3TC once daily
- DTG/TAF/3TC: Given as a single table containing 50mg DTG/25mg TAF/300mg 3TC once daily
- DRV/r: - Weight based dosing: ≥30kg - given 2 tablets each containing 400mg DRV/50mg ritonavir, ≥15 to <30Kg - given 1 tablet containing 600mg DRV PLUS one tablet containing 100mg ritonavir, ≥10 to <15Kg - given 1 tablet containing 400mg DRV/50mg ritonavir once daily
- TDF/3TC: Given as a single tablet containing 300mg TDF/300mg 3TC once daily
- TAF/3TC: Given as a single tablet containing 25mg TAF/300mg 3TC once daily
- TAF/FTC: Given as a single tablet containing 25mg TAF/200mg FTC once daily
- DTG:

>20kg:Given as a single tablet containing 50mg DTG

< 20kg:Given as a dispersible 10mg tablet for children as follows:

- 3-5.9 kg: 0.5 tablet
- 6-9.9kg:1.5 tablet
- 10-13.9kg: 2 tablets,
- 14-19.9kg:2.5 tablets,
- 20-24.9kg: 3 tablets(if above 20kg and cannot swallow a 50mg tablet or if 50mg tablets are not available.

Treatment of overdose: For this open-label study, any ARV intake exceeding the daily prescribed dose will be considered an overdose. An overdose will not be considered an adverse event (AE) unless it is accompanied by a clinical manifestation associated with the overdose. If the clinical manifestation presents with serious criteria, the event is a serious AE (SAE). Decisions regarding dose interruptions or modifications will be made by the investigator in consultation with the Chief Investigator based on the clinical evaluation of the participant. In the event of an overdose, the investigator or treating physician should closely monitor the participant for AEs/ SAE and document the quantity of the excess dose as well as the duration of the overdosing in the CRF.

Contraindications:

- DTG
  - Hypersensitivity to DTG or any component of the formulation
  - Coadministration with dofetilide
- DRV/r
  - Hypersensitivity to DRV/r or any component of the formulation
  - Severe (Child-Pugh class C) hepatic impairment
  - Coadministration with amiodarone, apixaban, lidocaine (systemic), rivaroxaban

Warnings and precautions:

- DTG
  - Hepatotoxicity: Hepatic adverse events, including elevated serum liver biochemistries, hepatitis, and acute liver failure, have been reported; these events have occurred in patients without underlying hepatic disease or other risk factors. Patients with hepatitis B or C may be at increased risk for worsening or development of increased transaminases; sometimes these increases were consistent with immune reconstitution syndrome or hepatitis B reactivation (particularly when anti-hepatitis therapy was withdrawn). Drug-induced liver injury has been reported with dolutegravir in combination with abacavir and lamivudine. Monitor patients for signs/symptoms of hepatotoxicity.
  - Hypersensitivity reactions: Rash, constitutional findings, and organ dysfunction (e.g., liver injury) have been reported. Discontinue immediately if signs of hypersensitivity (e.g., severe rash, rash with fever, malaise, fatigue, muscle/joint aches, blistering or peeling of skin, oral blisters/lesions, conjunctivitis, facial edema, hepatitis, eosinophilia, angioedema, difficulty breathing) occur. Monitor clinical status and liver function tests, and initiate supportive therapy as appropriate. If hypersensitivity occurs, do not reinitiate therapy with dolutegravir.
  - Immune reconstitution syndrome: Patients may develop immune reconstitution syndrome resulting in the occurrence of an inflammatory response to an indolent or residual opportunistic infection during initial HIV treatment or activation of autoimmune disorders (e.g., Graves disease, polymyositis, Guillain-Barré syndrome) later in therapy; further evaluation and treatment may be required.
- DRV/r
  - Fat redistribution: May cause redistribution/accumulation of fat (e.g., central obesity, buffalo hump, peripheral wasting, facial wasting, breast enlargement, cushingoid appearance)
  - Hepatotoxicity: Infrequent cases of drug-induced hepatitis (including acute and cytolytic) have been reported. Liver injury has been reported with use (including some fatalities), though generally in patients on multiple medications, with advanced HIV disease, hepatitis B/C coinfection, and/or immune reconstitution syndrome. Monitor patients closely; consider interrupting or discontinuing therapy if signs/symptoms of liver impairment occur
  - Hypersensitivity reactions: Protease inhibitors have been associated with a variety of hypersensitivity events (some severe), including rash, anaphylaxis (rare), angioedema, bronchospasm, erythema multiforme, Stevens-Johnson syndrome (rare), acute generalized exanthematous pustulosis, toxic epidermal necrolysis, and/or drug rash with eosinophilia and systemic symptoms (DRESS). Discontinue treatment if severe skin reactions develop. Severe skin reactions may be accompanied by fever, malaise, fatigue, arthralgias, hepatitis, oral lesions, blisters, conjunctivitis, and/or eosinophilia. Mild-to-moderate rash may occur early in treatment and resolve with continued therapy
  - Immune reconstitution syndrome: Patients may develop immune reconstitution syndrome resulting in the occurrence of an inflammatory response to an indolent or residual opportunistic infection during initial HIV treatment or activation of autoimmune disorders (e.g., Graves disease, polymyositis, Guillain-Barré syndrome, autoimmune hepatitis) later in therapy; further evaluation and treatment may be required
  - Sulfonamide allergy: Use with caution in patients with sulfonamide allergy (contains sulfa moiety)

Adverse reactions (reported as frequency >10%, grades ≥ 2, as part of combination ART):

- DTG
  - Gastrointestinal: Increased serum lipase (2% to 11%)
- DRV/r
  - Dermatologic: Skin rash (children and adolescents: 5% to 19%; adults: 6% to 7%)
  - Endocrine & metabolic: Increased LDL cholesterol (children and adolescents: grade 3: 3%; adults: 8% to 14%), increased serum cholesterol (children and adolescents: grade 3: 1%; adults: 1% to 25%), increased serum glucose (≤11%)
  - Gastrointestinal: Diarrhea (children and adolescents: 11% to 24%; adults: 9% to 14%), nausea (children and adolescents: 4% to 25%; adults: 4% to 7%), vomiting (children and adolescents: 13% to 33%; adults: 2% to 5%)

Drug interactions (Refer to the full prescribing information for important drug interactions with each ARV (attached)):

- DTG
  - Drugs that are metabolic inducers may decrease the plasma concentrations of DTG
  - DTG should be taken two hours before or six hours after taking cation-containing antacids or laxatives, sucralfate, oral supplements containing iron or calcium, or buffered medications. Alternatively, DTG and supplements containing calcium or iron can be taken together with food
- DRV/r
  - DRV/r is a CYP3A4 inhibitors and may increase serum concentrations of other drugs metabolized through this pathway and increase risk of toxicity
  - CYP3A4 inducers may decrease the serum concentration of DRV/r and increase risk of viral rebound
  - CYP3A4 inhibitors may increase the serum concentration of DRV/r and increase risk of toxicity

# 5.3 Prior and Concomitant Therapy

All medications (prescriptions or over-the-counter medications, vitamins, mineral supplements, herbal and naturopathic products) used during the study period must be documented in the CRF (dose, frequency, start and stop dates).

Any concomitant therapy that is contraindicated with any of the ARVs in a participant’s regimen, as described in the product monograph for each ARV, is prohibited during the study period.

# 5.4 Treatment Compliance

All participants will be asked to return unused medication and containers at month 1, 3, 6, 9 and 12. Treatment adherence will be evaluated using pill counts of unused study drugs and proportion or days covered (PDC) and recorded in the participants’ CRF. Poor adherence (defined as below 95%) will be reported to the Chief Investigator. Unused medications will be returned to the site pharmacist/designee for accountability and disposal.

Drug testing on DBS cards will be used to determine longer-term adherence to ARVs (over a period of 6 to 8 weeks). We will collect DBS cards for TFV-DP concentrations for retrospective analysis among RCT participants at months 1 and 6. Adherence based on DBS drug concentrations will be used to assess correlations with development of DRMs.

# 5.5 Provision of Treatment After the End of the Study

Upon exiting the trial after the month 12 visit, participants will remain on their current study regimen or change to a different regimen as determined by national HIV programs in each participating country, with recommendations provided by study investigators based on participant preference, most recent viral load results, adverse events, documented DRMs, known comorbidities and available formulations. The study will not supply ARVs to participants once they have exited the study and received their month 12 viral load results plus DRM results if applicable; all further ARVs will be supplied by the national ARV programs in each participating country.

# 6 Study Assessments and Procedures

This section describes the procedures and assessments that will be carried out in the study.

## 6.1 Time and Events Schedule

| **RCT**  Procedure | Months from enrolment | | | | | |
| --- | --- | --- | --- | --- | --- | --- |
|  | Screening / Enrollment (Day 1) | 1 | 3 | 6 | 9 | 12 |
| **Clinical and other assessments** | | | | | | |
| Written informed consent | x |  |  |  |  |  |
| Inclusion/exclusion criteria | x |  |  |  |  |  |
| Randomization | x |  |  |  |  |  |
| ART history | x |  |  |  |  |  |
| Medical history (past and current) | x |  |  |  |  |  |
| Concomitant medication | x | x | x | x | x | x |
| Smoking, alcohol and other drug use history | x | x | x | x | x | x |
| Vital signs | x | x | x | x | x | x |
| Physical examination | x | x | x | x | x | x |
| Height^1^ | x | x | x | x | x | x |
| Weight | x | x | x | x | x | x |
| Mid-upper arm circumference (MUAC) and Head circumference^2^ | x | x | x | x | x | x |
| Clinical screening for TB | x | x | x | x | x | x |
| Clinical assessment for opportunistic infections | x | x | x | x | x | x |
| Adverse events |  | x | x | x | x | x |
| Serious adverse events |  | x | x | x | x | x |
| Enhanced adherence counselling | x | x | x | x | x | x |
| Dispensing of medications | x | x | x | x | x | x |
| Collection of unused medications | x | x | x | x | x | x |
| Pill counts and PDC | x | x | x | x | x | x |
| Patient satisfaction using the HIV treatment satisfaction questionnaire (HIVTSQ) | HIVTSQ status version (HIVTSQs) |  |  | HIVTSQ change version (HIVTSQc) |  |  |
| **Laboratory investigations** | | | | | | |
| HIV-1 RNA viral load^3^ | x | x | x | x | x | x |
| Cr | x |  | x |  |  |  |
| AST | x |  | x |  |  |  |
| ALT | x |  | x |  |  |  |
| CD4 | x |  |  | x |  | x |
| CBC | x |  | x |  |  |  |
| DBS for TFV-DP levels |  | x |  | x |  |  |
| Drug resistance test^4^ |  |  | x | x | x | x |
| Urine pregnancy test^5^ | x | x | x | x | x | x |
| - 1. Height will be measured during each visit for participants aged 1 to 18 years old; for participants aged more than 18 years, it will be measured once during the screening/enrollment visit   2. MUAC and Head Circumference will be documented for children aged 3 to 5 years old   3. If the most recent VL from the cohort study was collected > 3 months, then do a repeat VL during the screening/enrollment visit   4. Genotypic drug resistance testing will be performed for all participants with viral load ≥ 200 copies/ml   5. For women of child bearing potential | | | | | | |

## 6.2 Assessments at Each Visit

The schedule of assessments is summarized in the Time and Events table (Section 6.1).

Written informed consent will be obtained at the start of the screening/enrolment visit before engaging in any study related evaluations and procedures.

All participants will complete the screening assessment of eligibility criteria on the same date as informed consent is obtained; if eligibility criteria are met, they will be enrolled into the study on the same day.

All participants will undergo enhanced adherence counseling sessions as well as an assessment for other reasons for viremia, such as drug-drug interactions, incorrect dosing, or poor absorption (as may be suggested by the presence of diarrhea and vomiting) starting at enrollment. Protocol-specified enhanced adherence counseling and assessment or management of other causes of viremia will continue for at least 3 sessions and will be carried out in person at the clinic and via phone calls where feasible. Additional enhanced adherence counseling will be performed for all participants with HIV-1 RNA > 200 copies/mL at follow up visits.

Follow up visits will take place at months 1, 3, 6, 9 and 12.

Follow up visits will take place within +/- 7 days of the specified visit schedule. Participants will be contacted by phone (phone call or text message) 24-48 hours before each appointment, and if they do not attend a scheduled visit, they will be contacted by phone the following day. If it is not possible to reach a participant by phone, then a home visit will be performed. The study clinician or study nurse/counsellor will also contact participants by phone at week 2 after the enrollment visit and any other time deemed necessary to follow up on adherence to study treatment, adverse events, or other study-related reasons. Additional/unscheduled visits are allowed as needed for clinical indications such as intercurrent illnesses, management of comorbidities, adverse events, or as desired by the participant.

In extenuating circumstances, such as travel restrictions or facility closures due to risk of COVID-19 infection or other unanticipated events, telephone calls may be used to complete as much information as possible on the case report forms (CRFs) for the scheduled study visits, and arrangements will be made for laboratory samples to be taken at or near the participant’s home and transported to one of the study laboratories for processing following the sample transportation and processing protocols.

In case of early termination, participants will be requested to complete a termination visit, including all assessments and laboratory investigations scheduled for the month 12 visit. Participants will be requested to attend the remaining study visits to month 12 even if they are no longer taking study medications.

HIV-1 RNA will be measured at months 1, 3, 6, 9 and 12. Other laboratory measures include DBS for TFV-DP levels, CD4 count, complete blood count, serum creatinine, ALT and AST per the Time and Events table (section 6.1)

Any participant whose HIV-1 RNA is ≥ 200 copies/mL at month 3, 6, 9 or 12 will have a reflex DRT performed. If DRT shows no new major DTG-associated or DRV/r-associated DRMs or is unsuccessful in sequencing the INSTI or PI region then they will have additional enhanced adherence counseling and will continue on their randomized regimen until the next follow-up viral load 3 months later, after which further action will be determined by the repeat viral load and DRT results.

Participants who have DRT showing ≥ 1 new major DTG-associated or DRV/r-associated DRM at month 3, or who have an increase in HIV-1 RNA ≥ 0.3 log from the most recent prior result at month 1 or 3 will be considered an early failure and will be withdrawn from the study; further management will be determined by the protocol-defined management algorithm (where there is no local algorithm, the study will develop one based on available information including treatment history, DRT and available ARVs) and they will be requested to attend all study visits until month 12.

Clinical measurements following the Time and Events Schedule (Section 6.1) will include:

- Height: Standing height will be measured to the nearest 0.5 cm barefoot, the back square against the wall tape, eyes looking straight ahead, with a set square resting on the scalp and against the wall (WHO, 1995)
- Weight: Weight will be assessed to the nearest 100 grams using a lever balance, barefoot, in light garments (WHO, 1995)
- Body Mass Index: The body mass index (BMI) will be calculated using the World Health Organization (WHO) formula as weight (in kilograms) divided by height (in meters) squared (WHO, 1995)
- Mid-upper arm circumference (MUAC): MUAC will be measured for children aged 3 to 5 years old in centimetres on a straight left arm midway between the tip of the shoulder and tip of the elbow using a MUAC tape
- Head circumference: will be measured for children aged 1 to 5 years old in centimetres using a flexible tape placed just above the eyebrows, above the pinnae and around the occipital prominence at the back of the head. The tape will be pulled gently to compress the hair and the circumference will be measured to the nearest 0.1cm
- Blood Pressure: Blood pressure will be measured using an automated blood pressure machine with the appropriately sized adult and pediatric blood pressure cuff around the mid arm with the participant sitting upright, both feet flat on the floor, with the back and arm supported (WHO, 1999)
- Patient satisfaction: We will use the HIV Treatment Satisfaction Questionnaire Status Version (HIVTSQs) to assess patient satisfaction at baseline and month 12. This is a 10-item questionnaire that is graded on a 6-point Likert scale with 6 being very satisfied and 0 being very dissatisfied. The 10 items look at overall satisfaction with treatment, control of HIV, side effects, demands of treatment, convenience, flexibility, understanding of treatment, lifestyle adjustment, whether they would recommend the same treatment to others and whether they would be happy to continue with the current treatment. We will use the HIV Treatment Satisfaction Questionnaire Change Version (HIVTSQc) at month 6 to assess for any change in satisfaction with change of medication.

## 6.3 Blood Sampling

Blood will be collected for laboratory investigations as outlined in the Time and Events Table (Section 6.1). Blood will be drawn from the antecubital fossa or any other appropriate site and collected in vacutainer tubes, and pipetted onto DBS cards. A total of up to 70 mL of blood will be collected from each study participant over the 1 year period as follows:

- Screening/enrolment visit: 15 mL of blood collected in three vacutainers
- Month 1: 10 mL of blood collected in two vacutainer tubes
- Month 3: 15 mL of blood collected in three vacutainer tubes
- Month 6: 10 mL of blood collected in two vacutainer tubes
- Month 9: 10 mL of blood collected in two vacutainer tubes
- Month 12: 10 mL of blood collected in two vacutainer tubes

All the standard procedures for performing these tests will be maintained to ensure results are of highest quality.

DBS cards for TFV-DP drug levels will be processed at the University of Cape Town in South Africa at the end of the study. Regulatory approval will be sought prior to shipment of the cards from each of the participating countries.

**6.3.1 Sample Processing in Kenya**

All blood samples collected from clinics across Kenya will undergo processing at the Kenya National Public Health Laboratory, including its associated regional testing facilities (KEMRI CGHR, KNH CCC, Kenyatta University Referral and Research Hospital, KEMRI Alupe, Migori County Referral Hospital, KEMRI Walter Reed, and AMPATH Care Laboratory. These facilities are fully ISO 15189 accredited, adhere to stringent internal and external quality control standards including a common interlaboratory comparison scheme, and are equipped to conduct all necessary tests for the study with a result turnaround time of ten working days. Samples will be transported to the testing laboratory following the guidelines outlined in the study's standard operating procedures manual ensuring that the sample chain of custody is maintained. Any leftover samples will be destroyed locally after analysis.

The Cobas 8800, Abbott m2000rt, Abbott Alinity, Hologic testing platforms, and Cepheid GeneXpert will be utilized to measure HIV viral load by assessing HIV-1 RNA levels. These machines will yield consistent results across the board because the machines are calibrated using quality control panels put across all machines to keep results consistent. Quality Control is done is done daily, in the morning before running samples. Any samples showing unsuppressed viral loads above 200 copies/ml will be transferred to the National Public Health Institute Genomics and Molecular Surveillance Laboratory. There, HIV drug resistance testing will focus on the entire Pol region using the Sanger 3730xl platform. Additionally, the Illumina MiSeq will be employed for whole-genome sequencing.

**6.3.2 Sample Processing in Mozambique**

All samples (plasma or plasma separation cards) collected within Maputo Province will be tested at the reference laboratory (Biotechnology and Genetics Laboratory of INS) located in Marracuene using COBAS 6800 (Roche Diagnostics). The reference laboratory is fully accredited (ISO 15189), uses internal controls, and participates in an external quality assurance control program for HIV VL testing.

All the samples collected in Beira Province will be processed by the Molecular Biology Laboratory in Ponta Gea which uses Allinity (Abbott) to test all the HIV VL samples. This laboratory also uses internal quality control and participates in an external quality control program.

All samples for sequencing will be shipped to the INS reference laboratory. These will be processed using Oxford Nanopore GridION (https://nanoporetech.com), which is a portable sequencer that identifies DNA bases by measuring the changes in electrical conductivity generated as DNA strands pass through a biological pore. A modified SARS-CoV-2 MIDNIGHT sequencing protocol for HIVDR using GridIon from Oxford Nanopore Technology will be used. The MIDNIGHT protocol is a modification of the ARTIC amplicon V3 sequencing protocol for MinION for nCoV-2019 developed by Josh Quick.

**6.3.3 Sample Processing in Tanzania**

Sample processing will be done at the Temeke Specialized Laboratory which is internationally accredited with ISO15189; 2022. The laboratory can process up to 4,200 viral load samples and 56 for drug resistance within 24 hours using Cobas 6800 and 8800 machines for viral load and Genetic analyser systems 3500 XL Applied Biosystem for drug resistance testing.

**6.3.4 Sample Processing in Lesotho**

In Lesotho, blood samples will be analysed by the respective site laboratories. The laboratories comply with national and international regulatory standards. In case viral load testing is not available on site, samples will be transported to the nearest testing laboratory by a dedicated motorbike transport service. Viral load testing will be done on the Roche COBAS 4800 platform.

Samples for DRT will be transported and analysed in South Africa given that there is currently no in-country capacity for resistance testing.

## 6.4 Qualitative Interviews

We will explore patient, provider, and policy maker experiences and beliefs around experiences of staying on vs. switching off DTG-containing ART. We will utilize a combination of in-depth interviews (IDIs), Key Informant Interviews (KII) and focus group discussions (FGDs) depending on the sampling group and topic, at baseline and months 6 and 12. We will use stratified convenience sampling to recruit participants already enrolled in the cohort and trial studies, stratified by potential factors such as site/country, sex/pregnancy status, age strata (noting caregivers of younger children will be sampled), viral suppression patterns, and more. Similarly, providers and policy makers will be recruited via stratified convenience sampling strategies. The IDIs, KIIs and FGDs will be conducted in-person or virtually, depending on the sampled group, in chosen languages by trained research staff, recorded in English, Sesotho or Portuguese and transcribed in English.

**Qualitative Study Population**

- PLWH on follow up in the HIV clinics in each site aged ≥ 10 years.
- Health care workers who participate in the care of people living with HIV.
- Policy makers, research scientists involved in HIV research, expert physicians-infectious disease specialists, virologists, physicians, pediatricians and technical advisors involved in the design and delivery of HIV services.

**Qualitative study Procedures**

***In-depth Interviews***

We will hold in-depth interviews with a maximum of 8 health care providers (2 per country) and 20 PLWH enrolled in Ndovu RCT (5 per country) or until no new themes emerge. Purposive sampling will be used to select interviewees. Healthcare providers who are actively involved in the care of PLWH in the sites where the study is being conducted will be invited to participate in the study. Those who consent will be taken through an interview virtually or physically at the convenience and availability of the interviewees.

Interview questions will explore themes around their experiences, perceptions and acceptability of switching versus remaining on DTG based regimens (appendix 7). We will invite participants randomized to both the DTG and DRV/r arms. Those selected for participation will be those who voluntarily consent to participate in the in-depth interviews and focus group discussions. In-depth interviews will last 1 hour and will focus on themes around their perspectives, preferences (acceptability) and experiences of either remaining on DTG-based regimens or switching to DRV-based regimens.

***Key Informant Interviews***

We will also approach 12 policymakers and experts who are involved in the design and delivery of HIV programs for key informant interviews to gain insights into their experiences, preferences and perspectives of managing DTG-based treatment failure: to switch or to remain of DTG-regimens. Policymakers will be technical advisors in the Ministry of Health, PEPFAR agencies and researchers actively involved in HIV research. These key informants will also be invited to give their insight on barriers and facilitators of switch to PI versus maintaining DTG in the management of DTG failure in a consultative stakeholder’s forum.

The socio-demographics of interviewees will be captured on REDCAP devoid of their identifiers. These socio-demographics will be linked to participants using participant IDs. Interviewees will provide consent for recording of interviews. The interviews will be held in person or virtually at the convenience of the interviewees by experienced qualitative research assistants who have been trained on the Ndovu study objectives and protocol. They will use a standardized open-ended interview protocol that has questions arranged in a logical flow to minimize confusion among participants (Appendix 7).

***Focus Group Discussion***

We will conduct focus groups among health care workers, PLWH, policy makers, technical advisors and experts in HIV care and treatment.

1. ***Health care workers Focus Groups***

We will conduct a total of 4 physical focus groups in each country, and one virtual focus group consisting of 4-6 Ministry of Health Officials or Heads of National Programs from each country participating the in study-Kenya, Tanzania, Mozambique and Lesotho The virtual focus group discussion will last 90 minutes. Participants will have provided infromed consent prior to joining the focus group discussion at a convenient time for all the participants. The focus group participants of the physical focus group will include a variety of health care providers such as medical doctors, clinicians, other ART prescribers, nurses, pharmacists and pharmaceutical technologists, and adherence support personnel. The focus group discussions will last 60-90 minutes, and all participants will have provided signed informed consent prior participation.

1. ***PLWH Focus Groups***

We will hold a maximum of five focus groups among PLWH who

are enrolled in the Ndovu RCT and consent to participate in focus group discussion. One of the focus groups will include participants who aged 10-19 years, while four of the focus groups discussion will include participants who are twenty years and above. Focus group discussions will be held in each of the countries the clinical trial is carried out in. The country with the highest number of Adolescents aged 10-19 years will hold the focus group discussion among Adolescents. Each focus group will have 8-10 participants. The group discussions will explore experiences, acceptability and satisfaction of remaining on DTG or switching to DRV-based regimens for management of DTG failure. The groups will have a heterogenous mix of PLWH who are randomized to remain on DTG or switch to DRV-based regimens.

We will hold focus groups until saturation is achieved, with a maximum of four focus groups. The focus group discussions will be held int the HIV clinics where the study is being carried out. Focus group discussions will be recorded for transcription.

1. ***Policy Makers***

We will hold a half-day consultative stakeholder’s forum or focus group where policy makers, technical advisors of HIV programs will sit together with health care workers to explore barriers and facilitators of managing treatment failure to DTG based regimens by either switching to DTG-based regimens or switching to DRV-based regimens. Barriers and facilitators of each scenario will be discussed with emphasis on either remaining or switching to DTG based regimens. Discussions from the stakeholder’s forum will be recorded and transcribed as the meeting goes on.

## 6.5 Clinical Evaluation

All clinical assessments conducted during each scheduled or unscheduled visit will be undertaken and reviewed by the study clinicians. Laboratory abnormalities that are clinically significant will be further evaluated to reach a diagnosis and guide clinical management. Abnormal laboratory values will be reported as AEs or SAEs as per section 8.

## 6.6 Samples for Future Research

There will be no samples stored for future research.

# 7 Statistical Methods

## 7.1 Analysis Populations

**Intent-to-Treat Exposed (ITT-E) Population**

The primary analysis for the primary outcome will use the Intent-to-Treat Exposed (ITT-E) study population, which is defined as all participants who receive at least one dose of study drug. Participants will be assessed according to the treatment regimen to which they were randomised.

**Per Protocol (PP) Population**

We will also analyze the primary endpoints using the per-protocol (PP) study population, which will exclude participants who switch treatments outside of the protocol recommendations during the course of the study.

**Safety Population**

Safety endpoints will be analyzed with the safety population, which is defined as all participants who were randomised to the trial arms.

## 7.2 Efficacy Analysis

The primary efficacy analysis will be conducted on the ITT-E study population. The difference in the proportion of participants with viral suppression 6-months after randomisation will be compared between the treatment arms stratified by the randomisation stratification factors. The point estimate for the difference between the treatment strategies will provide the best available data on the comparative efficacy of the two strategies in this study population.

We will also assess the superiority of switching to DRV/r-based therapy if the proportion with viral suppression is higher among those taking DRV/r-based therapy and the 95% confidence interval for the difference excludes no difference.

Should superiority of DRV/r-based therapy be demonstrated, we will include additional substantial-superiority analyses following a pre-specified series of ordered hypotheses.

Should DRV/r perform numerically better but not achieve statistical superiority, we would still be able to perform various informative analyses to identify:

- The subgroup of participants who do better on DTG or on DRV/r
- The ideal time to conduct DRT in patients failing to suppress on a DTG based regimen
- Appropriate patient selection for DRT
- Subpopulations of patients who may require switch to DRV/r without DRT
- DTG DRMs that predict suppression on DTG

## 7.3 Safety Analysis

Continuous variables will be summarized using means and standard deviations and medians (interquartile ranges) as appropriate, while categorical variables will be summarized using counts and percentages.

The following variables will be summarised:

- Suppression with HIV-RNA < 50 copies/mL at 12 months from randomization
- Suppression using HIV-RNA cut-off of < 50 copies/mL at 6 and 12 months from randomization
- Suppression using HIV-RNA cut-off of < 1,000 copies/mL at 6 and 12 months from randomization
- Suppression among different age strata (3-9 years, 10-19 years, ≥20 years)
- Suppression based on prior PI or INSTI exposure
- Suppression based on prior suspected or confirmed virologic failure on PI or INSTI
- Suppression based on participant sex
- Adherence levels, based on dried-blood spot TFV levels, associated with: suppression; development of treatment-emergent DRMs
- Incidence of treatment-emergent DRMs
- Patterns of accumulated DRMs
- Suppression based on pre-enrollment NRTI
- Suppression based on TDF vs TAF as the study NRTI

Change in CD4 from enrollment to RCT to month 6 will be summarized by treatment group using descriptive statistics, median (interquartile ranges). Wilcoxon Rank sum/median tests (as deemed appropriate after assessment of normality) shall be done to determine any significant difference in CD4 change between DTG-based regimen and DRV/r treatment groups to assess the impact of switching to PI-based.

Logistic regression models will be used to assess predictors of achieving suppression, development of DTG-associated DRMs (age, other DRMs, VL at switch to DTG, time with viremia, etc.) and development of opportunistic infections.

## 7.4 Qualitative Analysis

The primary goal of this analysis will be understanding key influences in experiences, treatment satisfaction and acceptability of remaining on DTG versus switch to DRV/r in the management of treatment failure among health care providers, policy makers and PLWH enrolled in Ndovu study. Interviews and focus group discussions will be recorded for transcription at the end of each day of data collection. Each transcript will be coded independently by two members of the study team. Transcripts and codes will be imported into ATLAS.ti version 7 (Scientific Software Development GmbH, Berlin, Germany) or Dedoose for data management and analysis. We will conduct content analysis to produce a description of key concepts and thematic analysis to analyze themes through a process of reading and re-reading transcripts, code development and refinement, code application to transcripts, discussion of code application, and revision of codes and code definitions.

## 7.5 Interim Analysis

A protocol defined interim analysis will take place once 242 participants (two-thirds of the study population) have data available on viral suppression at 3 months, defined by HIV VL < 200 copies/mL.

In order for the DSMB to recommend stopping the RCT due to established efficacy, the protocol defined interim analysis must demonstrate substantial superiority of PI-based therapy at a substantial superiority margin of 15%, beyond reasonable doubt. Beyond reasonable doubt refers to a Haybittle-Peto stopping rule (i.e. P<0.001), consequently no adjustment of the overall study type I error rate is required for the primary outcome analysis at 6-months.

The independent DSMB may recommend terminating either part of, or the entire study for safety or administrative reasons. Should the trial sponsor and Chief Investigator agree to terminate part or all of the study, a written statement fully documenting the reasons for such a termination will be provided to the ERCs and regulatory authorities within 15 days.

Potential reasons for stopping include:

- Established efficacy as demonstrated by the protocol defined interim analysis
- Clear evidence of increased risk of SAEs or deaths
  - The DSMB will review all data related to SAEs and deaths at regular intervals. If the DSMB identifies a discernable pattern of drug-related SAEs or deaths in the study they may recommend the study stop early.
- Futility
  - The study may be stopped for continued poor enrolment if it is deemed unlikely that the study will achieve a sufficient sample size to answer the primary hypotheses. Unless the study team provides convincing evidence that changes in recruitment procedures are likely to correct enrolment rates. The study is scheduled to enroll approximately 60 individuals per month. Poor enrolment is defined as enrolment of less than 1/3 of expected participants and will be assessed no sooner than nine months from study start date.
  - In the event of futility, DRT will be conducted on the Ndovu Cohort Study samples stored at baseline to identify baseline DRMs and enrich the pool of those potentially eligible for the RCT.

## 7.6 Software Used

Statistical analyses will be done using StataCorp. 2017 Stata Statistical Software: Release 15 College Station, TX: StataCorp LLC and the current version of R (currently version 4.4.0).

# 8. Adverse Events

The investigators and site staff personnel are responsible for detecting, documenting, and reporting events that meet the definition of an AE or SAE, with timely, accurate, and complete reporting and analysis of safety information.

## 8.1 Definition of an Adverse Event (AE)

An AE is any untoward medical occurrence in a participant administered a pharmaceutical product. It can be any unfavorable and unintended sign (including abnormal laboratory findings), symptom, or disease temporally associated with the use of the investigational product (IP) whether or not considered related to the IP. Clinically significant changes in physical examination or safety laboratory tests will be recorded as AEs.

AEs observed by the investigator, or reported by the participant, and any remedial action taken, will be recorded in the CRF and should be verifiable in the participant’s clinical notes throughout the study. The nature of each event, time of onset after drug administration, duration and severity will be documented together with the investigator’s opinion of the causal relationship to the investigational product (unrelated, unlikely, possible, probable, and definite).

All participants experiencing adverse events, whether considered associated with the use of the study medication or not, will be monitored until the symptoms subside and any clinically relevant changes in laboratory values have returned to baseline, or until there is a satisfactory explanation for the changes observed.

Procedures such as surgery should not be reported as AEs. However, the medical condition for which the procedure was performed should be reported if it meets the definition of an AE. Planned procedures such as surgery pre-planned prior to the participant’s enrolment into the study do not need to be reported as AEs if these are documented as planned at the screening visit.

Clinically significant changes in physical examination and blood safety profiles should also be recorded as AEs.

## 8.2 Definition of a Serious Adverse Event (SAE)

A serious adverse event (SAE) is defined as any untoward medical occurrence that meets any of the following conditions:

- Results in death
- Is life-threatening (the participant was at risk of death at the time of the event. It does not refer to an event that hypothetically might have caused death if it were more severe)
- Requires inpatient hospitalization or prolongs existing hospitalization
- Results in persistent or significant disability/incapacity
- Is a congenital anomaly/birth defect
- All possible drug-induced liver injury events with hyperbilirubinemia (defined as ALT ≥3xULN and bilirubin ≥ 2xULN (>35% direct) will be reported as SAEs

All SAEs must be reported to the Chief Investigator within 24 hours of a member of the study team becoming aware of the event. SAEs will be reported to the DSMB, ERCs, and regulatory authorities.

## 8.3 Assessment of Severity

All AEs will be graded using the Division of AIDS (DAIDS) Table for Grading the Severity of Adult and Pediatric Adverse Events, corrected version 2.1 – July 2017 Division of AIDS [41].

Note: There is a distinction between the seriousness and the severity of an adverse event. Severe is a measure of intensity; thus, a severe reaction is not necessarily a serious adverse event. For example, a headache may be severe in intensity but would not be classified as serious unless it meets one of the seriousness criteria for serious events.

## 8.4 Assessment of Causality

The relationship of each AE to the study drug will be assessed using the following definitions:

- Definite: distinct temporal relationship with drug treatment. Known reaction to agent or chemical group or predicted by known pharmacology. Event cannot be explained by participant’s clinical state or other factors
- Probable: reasonable temporal relationship with drug treatment. Likely to be known reaction to agent or chemical group or predicted by known pharmacology. Event cannot easily be explained by participant’s clinical state or other factors
- Possible: reasonable temporal relationship with drug treatment. Event could be explained by participant’s clinical state or other factors
- Unlikely: poor temporal relationship with drug treatment. Event easily explained by participant’s clinical state or other factors
- Unrelated: the event occurs prior to dosing. Event or intercurrent illness is due wholly to factors other than drug treatment

## 8.5 Collection and Follow-up of AEs

All AEs, regardless of seriousness, severity, or presumed relationship to study therapy, will be recorded using medical terminology in the adverse events case report form. Whenever possible, diagnoses will be given when signs and symptoms are due to a common etiology (e.g., cough, runny nose, sneezing, sore throat, and head congestion should be reported as “upper respiratory infection”). Investigators will record in the CRF their opinion concerning the relationship of the adverse event to study therapy (assessment of causality). All measures required for managing the adverse event will be recorded in individual participant charts.

The AE reporting period will be from consent until the participant’s final study visit. In addition, any untoward event that may occur subsequent to the reporting period that the Investigator assesses as possibly, probably or definitely related to the study drug medication will also be reported as an AE.

All AEs will be followed up until they are resolved or the participant’s participation in the study ends (i.e. until the final CRF is completed for that participant). In addition, all AEs and SAEs assessed by the Investigator as possibly related to the study drug will be followed even after the participant’s participation in the study is over. Such events will be followed until resolution, or until no further change can reasonably be expected. Deaths occurring more than 30 days after the final dose, which are considered to be unrelated to the study medication, will be reported as SAEs.

## 8.6 Methods of Detecting AEs and SAEs

Care must be taken not to introduce bias when detecting AEs and/or SAEs, particularly for an open-label trial. Open-ended and non-leading verbal questioning of the is the preferred method to inquire about AE occurrence. Questions that will be used in this study are:

- “How are you feeling?”
- “Have you had any (other) medical problems since your last visit/contact?”
- “Have you taken any new medicines, other than those provided in this study, since your last visit/contact?”

All abnormal laboratory investigation results will also be assessed as adverse events.

# 9 Data Handling

Participant data collected during the study will be logged in the study-specific electronic case report forms (eCRFs). To maintain confidentiality, the participant data will be identified only by protocol number, site number, participant number and clinic number.

## 9.1 Recording of Data

All study data will be logged in the appropriate eCRF by trained and authorized members of the study team. Such authorization will be given in writing by the Chief Investigator or as delegated through each Country Principal Investigator before the conduct of any study related activities. A delegation of authority log identifying who can enter data and/or sign off a CRF will be kept by the Chief Investigator and each Country Principal Investigator.

In case the eCRF is inaccessible, a paper CRF will be availed for use and will be scanned to produce an electronic copy within 24 hours. The eCRF will be kept updated by entering data within one week of any paper-based data collection.

The Study Monitor and Data Manager will review data on an ongoing basis and raise any discrepancies with site staff as required. The data will be reviewed and approved by the investigators following study completion of each.

## 9.2 Source Documentation and Study Records

The participant’s study identifier and recruitment date will be recorded in their study records. The following will also be documented in the study records: confirmation of written and oral consent, their clinical status, date of every study visit, date study medication was started and stopped, concomitant medications, copies of all relevant reports and laboratory tests and patient satisfaction using the standardized questionnaires, and comments on reports and documentation of any adverse events. All CRFs are attached (appendix 6).

## 9.3 Data Management

Data will be kept on a secure network drive at the University of Nairobi and only accessible to authorized personnel of the data management team and the study monitor. A log of authorized personnel will be kept by the Chief Investigator and each Country Principal Investigator.

## 9.4 Storage of Data

At the end of the study, participant records and other study documentation will be stored by the Chief Investigator as per Good Clinical Practice (GCP) standards. The investigator will put in place processes to prevent their accidental or premature erasure, destruction or disposal. In the event the Chief Investigator retires, relocates, or for other reasons withdraws from the duty of being in-charge of the study records, custody will be shifted to a co-investigator who will agree to take this responsibility. After the study is completed, and the final report has been prepared, all relevant paper documentation including CRFs will be archived. This will be done once the trial manager has determined that accessing the databases is no longer required for future analyses. After 15 years, databases will be pulled down. Physical copies of study documents such as informed consents will be shredded and re-shredded.

# 10 Quality Control and Quality Assurance

## 10.1 Monitoring Arrangements

The purpose of monitoring is to verify that the rights and wellbeing of human participants are protected; that trial data is accurate, complete and verifiable with source data; that the trial is conducted in compliance with the protocol, GCP and the applicable regulatory requirements.

The study monitor will conduct regular site visits for the purpose of monitoring various aspects of the study. The investigator must allow the study monitor, authorized representatives of the sponsor and authorized representatives of the regulatory authorities to inspect all CRF and corresponding source documents, e.g. original medical records, participant records and laboratory raw data, access to the clinical supplies, dispensing and storage areas and agree to assist with their activities if requested. The investigator will provide adequate time and space for monitoring visits.

The study monitor will query any missing or spurious data with the site data entry personnel, which should be resolved in a timely manner. A monitoring log will be maintained recording each visit, the reason for the visit, the monitor’s signature and investigator’s or designee’s confirmation signature.

## 10.2 Quality Assurance

In order to ensure the accuracy and reliability of data, all eCRFs will be completed immediately by the study staff conducting the relevant procedures, unless this would pose a hazard to the study participant. When paper forms are used, they will be entered into the eCRF within one week and then filed alongside other participant-specific hard copy documents in binders.

Written Standard Operating Procedures will be followed for all study activities to ensure the study is conducted and data is generated, documented and reported in compliance with the protocol, GCP and the applicable regulatory requirements.

All investigators and study personnel will maintain up-to-date GCP certification.

# 11 Ethical Considerations

## 11.1 Ethical Approvals

The protocol for this study, participant information and consent form, data collection tools and information about compensation available to participants, along with any other documentation required to fulfil Ethics Review Committees (ERC) obligations, will be submitted to the ERCs covering each study site as follows:

- Kenya:
  - Kenyatta National Hospital and University of Nairobi Ethics Review Committee (KNH-UoN ERC)
  - Jaramogi Oginga Odinga Teaching and Referral Hospital’s Institutional Scientific and Ethical Review Committee (JOOTRH ISERC)
- Tanzania:
  - Muhimbili University of Health and Allied Sciences Research Ethics Committee
  - National Health Research Ethics Committee
- Mozambique:
  - INS approval from the INS Scientific and Technical Review and the INS Institutional Review Board (CIBS-INS)
  - Comité Nacional de Bioética para Saúde (CNBS) - National Ethics Committee
- Lesotho:
  - National Health Research Ethics Committee

Any changes will be submitted as numbered and dated protocol amendments in accordance with ERC regulations.

## 11.2 Informed Consent

Potential participants ≥ 18 years will be provided with a verbal explanation of the nature of the study and a written participant information sheet provided.

The participant and/or guardian will be given adequate time for questions and clarifications before agreeing to participate. The investigator will explain the aims, methods, objectives and potential hazards of the study, as well as clarify to the participant that they are free to withdraw from the study at any time point for any reason without any detriment. In Kenya, informed consent will be administered in English, Kiswahili or Dholuo; while in Tanzania, this will be done in Kiswahili or English; in Mozambique, Portuguese; and in Lesotho, English and Sesotho languages will be used for informed consenting. Written informed consent will be obtained before the initiation of any study related evaluations and procedures. Any potential participant who is illiterate will choose an impartial witness (who is not a study team member); the witness must take part in the entire informed consent process, and the witness will counter-sign beside the participant’s thumbprint mark of agreement.

Written informed consent for participants aged 3 to 12 years will be provided by their caregivers or parents.

Participants aged 12 to 17 years who are accompanied by the caregivers will provide assent in addition to the written informed consent provided by their caregivers. This written assent only applies to adolescents aged 12 to 17 years who have been disclosed to about their HIV status and will be provided after being taken through what the study procedures entail in a simpler language that they are able to easily understand. They will sign or write initials against a thumbprint on an adolescent assent form. Their parents will have provided written informed consent. Participants aged more than 12 years of age and disclosure of HIV status has not been done will be enrolled in the RCT with the caregiver’s consent and the study team will work with the caregiver to support disclosure.

The Participant Information Sheets and Informed Consent forms are attached in Appendix 5 in the languages relevant to each country, which must be approved by the ERCs prior to use.

## 11.3 Study-specific Design Considerations

Risks from the study procedures and treatments, as well as the methods to reduce those risks are described in detail below.

1. The only invasive procedure we propose is phlebotomy. Phlebotomy will be performed by trained professional phlebotomists, clinicians or nurses employed by the study or by contracted study laboratories
2. Participants randomized to remain on DTG-based ART despite currently failing a DTG-based regimen with ≥ 1 major DTG-associated DRM may place that participant at higher risk of accumulating additional DRMs to INSTIs and NRTIs, which could compromise the efficacy of future treatment options. To reduce potential harm of treatment-emergent DRMs, the protocol includes close follow-up and early viral load monitoring and repeat DRTs that are more frequent than standard of care. The protocol also includes a definition of early failure as ≥ 0.3 log increase in HIV-1 RNA at month 1 or 3 compared to the most recent prior measure, or any treatment-emergent major DTG-associated or DRV/r-associated DRM at month 3. Any participant who meets criteria for early failure will be withdrawn from the study and be managed with a treatment algorithm following national guidelines and taking into consideration patient preference, ARV history, all DRT results, most recent viral load, comorbidities and available ARV formulations
3. Change in ARV regimen can be associated with new side effects or toxicities because of exposure to a new drug. There will be an ongoing assessment for any adverse drug events. Any adverse events will be graded using the Division of AIDS (DAIDS) Table for Grading the Severity of Adult and Paediatric Adverse Events (AEs) [41] (Appendix 4). Any patient experiencing a grade 3 or 4 AE or SAE will be reviewed by the study multidisciplinary team for appropriate action and reporting
4. Frequent and longer visits to the health facility could increase the risk of exposure to COVID-19 and other infectious diseases. The study will make every effort to minimize this by following all recommended precautions such as use of face masks, gloves and frequent cleaning of hands, ensuring that there is adequate space between people in the waiting areas, frequent and thorough cleaning of the clinic, minimizing contact time, and ensuring that any staff in the clinic who has any symptoms is not at work and receives treatment promptly.

## 11.4 Clinical Trial Insurance

All study participants will be insured against possible injury that might arise during the conduct of the study, with a valid policy by an insurer that is registered by the Insurance Regulatory Authority of Kenya or the respective insurance regulatory authorities of participating countries. The insurance will cover the liability of the investigator and sponsor, without excluding any damage that may be attributed to negligence.

## 11.5 Participant Compensation

Participants will be offered compensation for transportation costs associated with attending study visits, paid in local currency for each study site.

In Kenya, the level of reimbursement will be dependent on distance travelled to and from the clinic. If one is coming to the clinic from within a 15km radius of the study site, they will be reimbursed Kenya Shilling (KES) 400/= per visit. If coming from 15 to 25km of the study site, they will be reimbursed KES 600/= per visit. If coming from further away or if they require additional arrangements to attend a clinic visit e.g. a patient escort, they will be compensated based on the prevailing public transport rates and any costs related to the visit. All payments will be made to the participant at the end of the visit via mobile money transfer (MPESA). These rates will be reviewed regularly in the course of the study to cater for changes in transportation costs.

For Mozambique, 3.3 USD will be given to participants during each study visit to cater for travel and additional time needed at the clinic during study visits. This will be paid in cash.

In Lesotho, participants will receive travel reimbursements based on the distance travelled and road conditions. Reimbursements will range from 50 Lesotho Loti (LSL) for local residents to 200 LSL for those traveling longer distances. If travel to the facility requires a four-wheel-drive or multiple connections, higher costs will be reimbursed accordingly. Payments will be made via mobile money transfer (MPESA) or in cash for those without a phone.

In Tanzania, participants enrolled in Dar es Salaam will be compensated a total of 30,000 Tanzanian shillings for transport to and from the clinic and time spent at the clinic. This amount has been used in previous clinical trials and has been approved by the local community advisory board and the ethics committees. This will be paid in cash after the visit is complete.

Transport rates may be adjusted if local transportation costs change significantly during the study period.

## 11.6 Privacy of Personal Data

Only personal data required to investigate the primary and secondary objectives will be collected from the study participants. Data will be collected and handled with safeguards to maintain confidentiality and in conformity with data privacy and protection laws and regulations.

The investigators will ensure that the personal data will be:

- Processed fairly and lawfully
- Collected for specified, explicit, and legitimate purposes and not further processed in a way incompatible with these purposes
- Adequate, relevant, and not excessive in relation to said purposes
- Accurate and, where necessary, kept current

Written consent for the processing of personal data will be obtained from the participant before collection of data. The participant has the right to access their personal data through the investigator and request alteration of any data that is inaccurate or incomplete. Adequate technical and organizational processes will be in place to prevent unauthorized access, inappropriate disclosures, accidental or unlawful destruction, or accidental loss or alteration. Investigators and study team members with access to personal data will keep the identity and participant information confidential.

Study staff will also endeavour to maintain privacy and confidentiality in all interactions with study participants. Any contacts via phone will be done without identifying the study participant’s HIV status or that they are participating in a research study to other individuals answering the phone.

Specific measures to ensure secure storage of data will be implemented. All study data will be entered in electronic CRFs on password- or biometric-protected tablets or laptops. Only study staff will have access to these tablets or laptops. Personally identifiable data will be stored distinctly separate from clinical data in password-protected and encrypted files. Any time paper-based CRFs are required they will be locked in a separate filing cabinet only accessible to the study staff. Analytic datasets will not contain personally identifiable information, and in these datasets, study participants will be identified only by a coded study identifier. The key to the coded study identifier will be stored in a password-protected and encrypted file.

## 11.7 Independent Data Safety Monitoring Board

The Data Safety Monitoring Board (DSMB) will monitor the main safety and efficacy outcome measures and the overall conduct of the trial, with the aim of safeguarding the wellbeing and interests of the study participants.

The DSMB will consist of 5 members, each of whom brings one or more of the following qualifications: biostatistician with experience in clinical trials; physician with HIV therapeutics expertise; HIV pharmacist or clinical pharmacologist; investigator with expertise in clinical trials conduct and methodology. DSMB members will not participate in the study as investigators, nor will they have conflicts of interest regarding the study, institutions conducting the trial, study sponsor, the study drug being tested, or any other activity or entity that might affect their objectivity. Potential DSMB members will be asked to declare conflicts of interest before participation.

Ad-hoc specialists may be requested to participate as non-voting members from time to time if additional expertise is desired, as determined by the DSMB Chair.

The specific membership, frequency and methods of the DSMB, and the study aspects to be reviewed, are outlined in the DSMB Charter (appendix 10).

# 12. Administrative Procedures

## 12.1 Protocol Modifications

This protocol will not be modified without approval of a formal amendment. Protocol amendments will not be implemented before ERC approval, or when the relevant competent authority has raised any grounds for non-acceptance, except when necessary to address immediate dangers to participants. In the latter case, the modifications will be promptly presented to the ERCs. When the alteration(s) relate only to logistic or administrative aspects of the study, the ERCs will only be notified.

Where data recorded in the eCRF and source document depart from the protocol, this will be reported as a protocol deviation or violation. These will be documented on a protocol deviations or violations log describing this departure, the circumstances requiring or resulting in it, and measures taken to avoid recurrence. All protocol deviations and violations will be reported to the ERCs.

## 12.2 Regulatory Notification

The study protocol and all requisite documents will be approved by the relevant regulatory authority in each country prior to study initiation in that country:

- Kenya
  - Kenyatta National Hospital and University of Nairobi Ethics Review Committee (KNH-UoN ERC)
  - Jaramogi Oginga Odinga Teaching and Referral Hospital’s Institutional Scientific and Ethical Review Committee (JOOTRH ISERC)
  - The Pharmacy and Poisons Board of Kenya (PPB)
  - The National Commission for Science, Technology and Innovation (NACOSTI)
- Tanzania
  - Institutional Review Board at Muhimbili University of Health and Allied Sciences
  - National Health Research Ethics Committee
  - Tanzania Medicines and Medical Devices Authority (TMDA)
- Mozambique
  - INS approval from the INS Scientific and Technical Review and the INS Institutional Review Board (CIBS-INS)
  - Comité Nacional de Bioética para Saúde (CNBS) - National Ethics Committee
- Lesotho
  - National Health Research Ethics Committee

All SAEs and protocol deviations or violations will be reported to the respective site and country ERCs and regulatory bodies. Notification of early termination of the study will be done within 15 days. When the study is complete, a preliminary report will be submitted within 30 days and a final report within 180 days.

## 12.3 Publication Policy

Following completion of the study, the findings of the research are planned for oral presentation at a scientific conference and publication in a scientific journal. The policy of the International Committee on Medical Journal Editors (ICMJE) member journals will be adhered to. Interim analysis during the follow-up period may be submitted for presentation or publication if deemed of scientific value by the Chief Investigator.

The Chief Investigator and co-investigators will formulate plans, organize data and prepare manuscripts for publication. Relationships with funding organizations, any other potential conflicts of interest, and the role of the sponsor will be disclosed in all manuscript submissions. Funding organizations will have the opportunity to review and comment on conference abstracts and manuscripts before submission but will not be involved in the decision to publish. All co-authors will have access to all data, and the Chief Investigator will have final decision on publication.

## 12.4 Drug Accountability

The investigator will ensure that the study drugs are only used in accordance with the protocol. Drug supplies will be kept in a secure, limited access storage area under the recommended storage conditions accessible only to those authorized by the investigator to dispense to eligible participants. The investigator will ensure that records are maintained showing the receipt and disposition of all study supplies.

## 12.5 Sample Shipment Processing

All samples will be transported and processed following the standard operating procedures in the laboratory manual.

In Kenya, Tanzania and Mozambique all samples except DBS for TFV-DP levels will be processed locally. Samples for DRT in Lesotho will be shipped to South Africa for testing.

DBS cards for TFV-DP levels will be shipped to the University of Cape Town at the end of the study for testing. The material transfer agreement and approval for shipment of samples to South Africa will be sought from the country regulatory authorities.

All samples sent to the laboratory will be de-identified; each sample will be allocated a research number. Patient names and other potentially identifying information will be removed. All samples will be destroyed once analysed according to international laboratory standards and GCLP guidelines. Samples will be used strictly for the purposes of the study and there will be no secondary data mining by the laboratory or other researchers.

# 13 References

1. The State of HIV Treatment, Testing, and Prevention in Low- and Middle-Income Countries; HIV Market Report 2023. Boston, MA, USA: Clinton Health Access Initiative, **2023**.

2. Consolidated Guidelines on HIV Prevention, Testing, Treatment, Service Delivery and Monitoring: Recommendations for a Public Health Approach. Geneva: World Health Organization, **2021**.

3. Kenya HIV Prevention and Treatment Guidelines. 2022 ed. Nairobi: National AIDS/STI Control Program, Ministry of Health Kenya, **2022**.

4. HIV care guide for adults, pregnant teenagers, lactating mothers and children: National STI, HIV and AIDS Control Program, National Directorate of Public Health, Ministry of Health Mozambique, **2023**.

5. National guidelines for the management of HIV and AIDS: National AIDS Control Programme, Ministry of Health, Community Development, Gender, Elderly and Children of Tanzania, **2019**.

6. ART clinical guidelines for the management of HIV in adults, pregnancy and breastfeeding, adolescents, children, infants and neonates, version 4: Rebuplic of South Africa National National Department of Health, **2023**.

7. Chu C, Tao K, Kouamou V, et al. Prevalence of Emergent Dolutegravir Resistance Mutations in People Living with HIV: A Rapid Scoping Review. Viruses **2024**; 16(3): 399.

8. Brown J, Ringera I, Luoga E, et al. GIVE MOVE: Randomized Trial on Genotype-Informed Management of Viremia in Children and Adolescents. Conference on Retroviruses and Opportunistic Infections. Denver, USA, **2024**.

9. Bello G, Pals S, Bighignoli B, et al. Emerging Dolutegravir Resistance Among Children Being Investigated for Treatment Failure in Malawi. Conference on Retroviruses and Opportunistic Infections. Denver, USA, **2024**.

10. Ismael N. HIV drug resistance profile in clients experiencing treatment failure after the transition to dolutegravir-based first-line antiretroviral treatment in Mozambique. International Workshop on HIV Drug Resistance and Treatment Cape Town, South Africa, **2023**.

11. Kingwara L, Onwonga V, Madada R, et al. Dolutegravir Resistance in Resource-Limited Settings. Conference on Retroviruses and Opportunistic Infections. Denver, USA, **2024**.

12. Bosch B, Sokhela S, Akpomiemie G, et al. High rates of long-term HIV RNA re-suppression after virological failure on dolutegravir in the ADVANCE trial. 12th IAS Conference on HIV Science. Brisbane, Australia, **2023**.

13. Tao K, Rhee SY, Chu C, et al. Treatment Emergent Dolutegravir Resistance Mutations in Individuals Naive to HIV-1 Integrase Inhibitors: A Rapid Scoping Review. Viruses **2023**; 15(9).

14. Akil B, Blick G, Hagins DP, et al. Dolutegravir versus placebo in subjects harbouring HIV-1 with integrase inhibitor resistance associated substitutions: 48-week results from VIKING-4, a randomized study. Antivir Ther **2015**; 20(3): 343-8.

15. Castagna A, Maggiolo F, Penco G, et al. Dolutegravir in antiretroviral-experienced patients with raltegravir- and/or elvitegravir-resistant HIV-1: 24-week results of the phase III VIKING-3 study. J Infect Dis **2014**; 210(3): 354-62.

16. Eron JJ, Clotet B, Durant J, et al. Safety and efficacy of dolutegravir in treatment-experienced subjects with raltegravir-resistant HIV type 1 infection: 24-week results of the VIKING Study. J Infect Dis **2013**; 207(5): 740-8.

17. Naeger LK, Harrington P, Komatsu T, Deming D. Effect of dolutegravir functional monotherapy on HIV-1 virological response in integrase strand transfer inhibitor resistant patients. Antivir Ther **2016**; 21(6): 481-8.

18. Paton NI, Musaazi J, Kityo C, et al. Dolutegravir or Darunavir in Combination with Zidovudine or Tenofovir to Treat HIV. N Engl J Med **2021**; 385(4): 330-41.

19. Kanise H, van Oosterhout JJ, Bisani P, et al. Virological Findings and Treatment Outcomes of Cases That Developed Dolutegravir Resistance in Malawi's National HIV Treatment Program. Viruses **2023**; 16(1).

20. Boyd MA, Moore CL, Molina JM, et al. Baseline HIV-1 resistance, virological outcomes, and emergent resistance in the SECOND-LINE trial: an exploratory analysis. Lancet HIV **2015**; 2(2): e42-51.

21. Group S-LS, Boyd MA, Kumarasamy N, et al. Ritonavir-boosted lopinavir plus nucleoside or nucleotide reverse transcriptase inhibitors versus ritonavir-boosted lopinavir plus raltegravir for treatment of HIV-1 infection in adults with virological failure of a standard first-line ART regimen (SECOND-LINE): a randomised, open-label, non-inferiority study. Lancet **2013**; 381(9883): 2091-9.

22. La Rosa AM, Harrison LJ, Taiwo B, et al. Raltegravir in second-line antiretroviral therapy in resource-limited settings (SELECT): a randomised, phase 3, non-inferiority study. Lancet HIV **2016**; 3(6): e247-58.

23. Paton NI, Kityo C, Hoppe A, et al. Assessment of second-line antiretroviral regimens for HIV therapy in Africa. N Engl J Med **2014**; 371(3): 234-47.

24. Paton NI, Kityo C, Thompson J, et al. Nucleoside reverse-transcriptase inhibitor cross-resistance and outcomes from second-line antiretroviral therapy in the public health approach: an observational analysis within the randomised, open-label, EARNEST trial. Lancet HIV **2017**; 4(8): e341-e8.

25. Paton NI, Musaazi J, Kityo C, et al. Efficacy and safety of dolutegravir or darunavir in combination with lamivudine plus either zidovudine or tenofovir for second-line treatment of HIV infection (NADIA): week 96 results from a prospective, multicentre, open-label, factorial, randomised, non-inferiority trial. Lancet HIV **2022**; 9(6): e381-e93.

26. Arribas JR, Pozniak AL, Gallant JE, et al. Tenofovir disoproxil fumarate, emtricitabine, and efavirenz compared with zidovudine/lamivudine and efavirenz in treatment-naive patients: 144-week analysis. J Acquir Immune Defic Syndr **2008**; 47(1): 74-8.

27. Gallant JE, DeJesus E, Arribas JR, et al. Tenofovir DF, emtricitabine, and efavirenz vs. zidovudine, lamivudine, and efavirenz for HIV. N Engl J Med **2006**; 354(3): 251-60.

28. Anderson PL, Liu AY, Castillo-Mancilla JR, et al. Intracellular Tenofovir-Diphosphate and Emtricitabine-Triphosphate in Dried Blood Spots following Directly Observed Therapy. Antimicrob Agents Chemother **2018**; 62(1).

29. Castillo-Mancilla JR, Zheng JH, Rower JE, et al. Tenofovir, emtricitabine, and tenofovir diphosphate in dried blood spots for determining recent and cumulative drug exposure. AIDS Res Hum Retroviruses **2013**; 29(2): 384-90.

30. Castillo-Mancilla JR, Morrow M, Coyle RP, et al. Tenofovir Diphosphate in Dried Blood Spots Is Strongly Associated With Viral Suppression in Individuals With Human Immunodeficiency Virus Infections. Clin Infect Dis **2019**; 68(8): 1335-42.

31. Dorward J, Govender K, Moodley P, et al. Urine tenofovir and dried blood spot tenofovir diphosphate concentrations and viraemia in people taking efavirenz and dolutegravir-based antiretroviral therapy. AIDS **2024**; 38(5): 697-702.

32. van Heerden JK, Meintjes G, Barr D, et al. Relationship Between Tenofovir Diphosphate Concentrations in Dried Blood Spots and Virological Outcomes After Initiating Tenofovir-Lamivudine-Dolutegravir as First-Line or Second-Line Antiretroviral Therapy. J Acquir Immune Defic Syndr **2024**; 95(3): 260-7.

33. Havens JP, Bares SH, Lyden E, et al. Effectiveness and Safety of Bictegravir/Emtricitabine/Tenofovir Alafenamide in Patients With HIV-1 Infection and Ongoing Substance Use Disorder: The BASE Study. Open Forum Infect Dis **2023**; 10(3): ofad080.

34. Jennings L, Ferraris CM, Castillo-Mancilla JR, et al. Comparing Predictive Ability of Two Objective Adherence Measures in a Community-Based Cohort on Antiretroviral Therapy in South Africa: Tenofovir Diphosphate Concentrations and Electronic Adherence Monitors. J Acquir Immune Defic Syndr **2023**; 93(4): 327-32.

35. Coyle RP, Morrow M, Mann SC, et al. Tenofovir-Diphosphate and Emtricitabine-Triphosphate Adherence Benchmarks in Dried Blood Spots for Persons with HIV Receiving Tenofovir Alafenamide and Emtricitabine-based Antiretroviral Therapy (QUANTI-TAF). Clin Infect Dis **2024**.

36. Jennings L, Robbins RN, Nguyen N, et al. Tenofovir diphosphate in dried blood spots predicts future viremia in persons with HIV taking antiretroviral therapy in South Africa. AIDS **2022**; 36(7): 933-40.

37. Morrow M, MaWhinney S, Coyle RP, et al. Predictive Value of Tenofovir Diphosphate in Dried Blood Spots for Future Viremia in Persons Living With HIV. J Infect Dis **2019**; 220(4): 635-42.

38. Singh Y, Castillo-Mancilla J, Madimabe R, et al. Tenofovir diphosphate in dried blood spots and HIV-1 resistance in South Africa. AIDS Res Ther **2023**; 20(1): 67.

39. Human Immunodeficiency Virus-1 Infection: Developing Antiretroviral Drugs for Treatment: U.S. Department of Health and Human Services, **2015**.

40. Marley-Zagar E, White IR, Royston P, Barthel FM, Parmar MKB, Babiker AG. artbin: Extended sample size for randomized trials with binary outcomes. Stata J **2023**; 23(1): 24-52.

41. Division of AIDS (DAIDS) Table for Grading the Severity of Adult and Pediatric Adverse Events, Corrected Version 2.1. In: Services UDoHaH. Bethesda, MD: National Institute of Allergy and Infectious Diseases, **2017**.

# 14 Protocol Signature Page

I agree to conduct the trial in accordance with GCP and the applicable regulatory

requirements and with the approved protocol.

I agree to comply with the procedures for data recording / reporting.

I agree to permit monitoring, auditing and inspection and to retain the trial related

essential documentation for the period of time required according to ICH-GCP.

Name of Chief Investigator: Loice Achieng Ombajo

Signature:

Date:

Country Principal Investigators:

| Country | Name | Signature | Date |
| --- | --- | --- | --- |
| Mozambique | Nalia Ismael |  |  |
| Tanzania | Patricia Munseri |  |  |
| Lesotho | Irene Ayakaka |  |  |

# 15 Appendices

Appendix 1: Participating Study Sites

Appendix 2: Study Flowchart

Appendix 3: Study Timeline

Appendix 4: Division of AIDS Table for Grading the Severity of Adult and Paediatric Adverse Events (DAIDs) Tables

Appendix 5: Participant Information Sheet and Consent Forms (ICFs)

Appendix 6: Case Report Forms (CRFs)

Appendix 7: Qualitative Interview and Focus Group Discussions Guides

Appendix 8: Research Team and Qualifications

Appendix 9: Investigators Documents

Appendix 10: Data and Safety Monitoring Board Charter

Appendix 11: Steering Committee Charter

Appendix 12: Study Drug Information

Appendix 13: Letters of Support

Appendix 14: Confirmation of Funding

Appendix 15: Study Budget and Narrative
