# Supplementary material for: The dolutegravir failure cohort: A multi-country longitudinal cohort with a randomised clinical trial of continued dolutegravir versus switch to darunavir in people with viraemia while on dolutegravir in Sub-Saharan Africa (The Ndovu Study) protocol
Source: PLoS One. 2026 Mar 13;21(3):e0330792. doi: 10.1371/journal.pone.0330792 (PMC12987441; doi:10.1371/journal.pone.0330792)
Supplement: S6 File — (PDF) [file pone.0330792.s006.pdf]

Ndovu Steering Committee Charter

Investigating the optimal management of dolutegravir resistance

Short Title: Ndovu Study

Sponsor: University of Nairobi

This is a collaborative study between the University of Nairobi, acting as Sponsor, and Instituto Nacional de Saúde (INS), Muhimbili University of Health and Allied Sciences (MUHAS), SolidarMed, and the London School of Hygiene and Tropical Medicine (LSHTM)

Funding: Bill & Melinda Gates Foundation

Version 1.4: 17-March-2025

Investigators

Chief Investigator

Loice Achieng Ombajo, MBChB, M.Med, DLSHTM, MSc (ID), FRCP

Principal Investigators

Principal Investigator, Mozambique: Nalia Ismael, BSc, MSc, PhD
Principal Investigator, Tanzania: Patricia Munseri, MD, MMed, MPH, PhD
Principal Investigator, Lesotho: Irene Ayakaka, MBChB, MPH, MRes

Co-Investigators

Co-investigators – Kenya:

Jeremy Penner, MD, MHSc, DTM&H, CCFP, FCFP
Emily Wangui Kamau, MBChB, M.Med, MSc (ID)
Patrick Amoth, MBChB, M.Med
Andrew Mulwa, MBChB, Msc
Elizabeth Abong'o, KRCHN
Leonard Kingwara, BSc, MPH, PhD
Dalton C. Wamalwa, MBChB, M.Med, MPH
James Wagude, MBChB, M.Med
Rose Wafula, MBChB, MPH
Lazarus Momanyi, MBChB, MPH
Joseph Nkuranga, MBChB, MSc (Epi)
Florentius Ndinya, MBChB, M.Med,
Anne-Marie Macharia, MBChB, M.Med
Simon Wahome, BPharm, MPharm
Anthony Kiplagat, DCM, BA, MSc
Caroline Wafula, BPharm, MPharm, MBA
Lisa Abuogi, BA, MD, Res, MSc
Rena Patel, BA, M Phil, MD, MPH

Co-Investigators – Mozambique:

Raquel Matavele Chissumba, BSc, MSc, PhD
Patricia Maria Ramgi, MD

Co-Investigators – Tanzania:

Muhammad Bakari, MD, M.Med, PhD
Jamila Said Didi, MD, MBA, M.Med, MSc (Nephrology)

Co-Investigators – Lesotho:

Niklaus Labhardt, MD, MIH, FMH
Anna Klicpera, MD, DTM, MSc
Tapiwa Tarumbiswa MBBS, MBA

Co-Investigators – London School of Hygiene and Tropical Medicine (LSHTM):

Daniel James Grint, BSc, MSc, PhD
Charles Opondo, BPharm, MSc, PhD

1. Introduction

This charter is for the Investigating the optimal management of dolutegravir resistance, covering both the Ndovu cohort study and the Ndovu randomized controlled trial (RCT).

The charter is intended to be a living document. The Ndovu Steering Committee (NSC) may wish to review it at regular intervals to determine whether any changes in procedure are needed.

2. Responsibilities of the NSC

The role of the NSC is to provide oversight and advice, through its Chair, to the Chief Investigator and the Trial Coordinating Team on all appropriate aspects of the project. In particular, the NSC will focus on the study's design, progress, adherence to the protocol and good clinical practice standards, patient safety, and consideration of new information. The NSC will i) monitor the progress of both the Ndovu Cohort Study and Ndovu RCT, ii) review relevant literature, research work and guidelines that may inform the conduct of the study, iii) consider recommendations of the data safety and monitoring board (DSMB), and iv) advise on possible changes to the conduct of the study for consideration by the study team such as amendment of the study protocols where this is necessary.

In particular, the Chair of the NSC will have additional responsibilities including:

- Liaising with the Chief Investigator to schedule NSC meetings and prepare the meeting agenda
- Providing an independent, experienced opinion if conflicts arise between the research team, the funder, or any of the participating organisations, collaborators or agencies
- Leading the NSC to provide regular, impartial oversight of the study, especially to identify and pre-empt problems
- Ensuring that protocol amendments are debated and endorsed by the NSC before implementation
- Being available to provide independent advice as required
- Commenting in detail (when appropriate) regarding NSC recommendations that may affect the duration or cost of the study including extension or termination of the study
- Overseeing the timely analysis, writing up and publication of the main trial results. The independent members of the NSC will have the opportunity to read and comment on the proposed main publications of trial data prior to submission

3. Membership

The NSC will consist of 6 independent members comprising technical experts in HIV care and a patient community representative and the study members including the lead investigators, statisticians, study coordinators and management team. The team has qualifications and experience in HIV treatment, clinical research, guidelines and policies development, and stakeholder engagement.

NSC members will not participate in the study as investigators, nor will they have conflicts of interest regarding the study, institutions conducting the study, study sponsor, the study drug being tested, or any other activity or entity that might affect their objectivity.

Ad-hoc specialists may be invited to participate as non-voting members at any time if additional expertise is desired, as determined by the NSC Chair.

The NSC members are:

Name	Affiliation
**Independent members**	
Robert W Shafer	Stanford Medicine
Irene Mukui	University of Washington
Michael Jordan (Chair)	World Health Organization
Mike Reid	Presidential Emergency Plan for AIDS Relief (PEPFAR)
Seth Inzaule	World Health Organization
Juliana de Fatima da Silva	US Centers for Disease Control and Prevention
Dorothy Onyango	Women Fighting HIV and AIDS in Kenya (WOFAK)
**Non-independent members**	
Loice Achieng Ombajo	Chief Investigator, University of Nairobi, Kenya
Nalia Ismael	Principal Investigator, INS, Mozambique
Patricia Munseri	Principal Investigator, MUHAS, Tanzania
Irene Ayakaka	Principal Investigator, SolidarMed, Lesotho
Joseph Nkuranga	Study Coordinator, University of Nairobi, Kenya
Jeremy Penner	Co-Investigator, University of Nairobi, Kenya
Emily Kamau	Study Physician, University of Nairobi, Kenya
Victor Omodi	Study Statistician, University of Nairobi, Kenya
Daniel Grint	Lead Statistician, LSHTM, United Kingdom

4. Tasks and Expected Time Commitment

The Ndovu Study is expected to run for about 24 months, with a 6-month recruitment period for both the cohort study and RCT. Ndovu Cohort Study participants will be followed up for at least 12 months. Ndovu RCT will be recruited from the cohort and will be followed up for 12 months.

Before the start of study, NSC members will review the study protocols for any major concerns (approximately 2 hours).

During the conduct of the study, the NSC members will participate in five pre-planned teleconference meetings plus ad-hoc meetings as needed, and develop recommendations during each meeting (each meeting will take approximately 1.5 hours). The meetings are tentatively scheduled to start in May 2025 and will be scheduled six-monthly thereafter until May 2027.

Prior to each meeting, NSC members will review summary reports prepared by the Chief Investigator and Trial Coordinating Committee (approximately 1 hour, within one week before each meeting).

5. Meeting Procedures

NSC meetings will be convened by the Chair of the NSC and held at least 6-monthly. Each meeting will require a quorum of 4 independent NSC members in addition to the non-independent members. Additional attendees to NSC meetings may include:

- Ad-hoc specialists invited by the NSC Chair as needed

An agenda for each meeting will be shared a week prior to the meeting along with NSC progress reports.

During each meeting, the Chief Investigator will be invited to share a brief report addressing the reports that had been shared with members. This will be followed by deliberations on any highlighted or emerging issues.

The decisions and recommendations by the NSC should be reached by consensus. If consensus cannot be reached then a vote may be taken.

Meetings will be recorded and minutes taken by the Trial Manager or Coordinator.

6. Reports Provided to the NSC

The Chief Investigator is responsible for ensuring the NSC receives the following reports:

- Previous NSC meeting report and updates on action items
- Study progress report including:
- DSMB meeting reports
- Regulatory audit reports

Additionally, any relevant recent publications that may affect the conduct of the study will be shared by the Chief Investigator or any member of the NSC through the Chair.

7. Reports from the NSC

The Chair of the NSC is responsible for preparing a summary report from each NSC meeting. This report will be submitted to the Chief Investigator within 4 weeks of the meeting.

Agreement

I agree to be on the Steering Committee for the study titled "Investigating the optimal management of dolutegravir resistance (Ndovu Study)" and I am in agreement with the content of this Steering Committee Charter. I will keep all information gathered during the Steering Committee meetings strictly confidential.

____________________	____________________	____________________
Name	Signature	Date
