# Supplementary material for: The dolutegravir failure cohort: A multi-country longitudinal cohort with a randomised clinical trial of continued dolutegravir versus switch to darunavir in people with viraemia while on dolutegravir in Sub-Saharan Africa (The Ndovu Study) protocol
Source: PLoS One. 2026 Mar 13;21(3):e0330792. doi: 10.1371/journal.pone.0330792 (PMC12987441; doi:10.1371/journal.pone.0330792)
Supplement: S7 File — (PDF) [file pone.0330792.s007.pdf]

Appendix 10: Data and Safety Monitoring Board Charter

Investigating the optimal management of dolutegravir resistance: an open-label randomised controlled trial of maintaining dolutegravir or switch to ritonavir-boosted darunavir

Short Title: Ndovu RCT

Sponsor: University of Nairobi

This is a collaborative study between the University of Nairobi, acting as Sponsor, and Instituto Nacional de Saúde (INS), Muhimbili University of Health and Allied Sciences (MUHAS), SolidarMed, and the London School of Hygiene and Tropical Medicine

Funding: Bill & Melinda Gates Foundation

Version 1.2: 24-February-2025

Investigators

Chief Investigator

Loice Achieng Ombajo, MBChB, M.Med, DLSHTM, MSc (ID), FRCP

Principal Investigators

Principal Investigator, Mozambique: Nalia Ismael, BSc, MSc, PhD
Principal Investigator, Tanzania: Patricia Munseri, MD, MMed, MPH, PhD
Principal Investigator, Lesotho: Irene Ayakaka, MBChB, MPH, MRes

Co-Investigators

Co-investigators – Kenya:

Jeremy Penner, MD, MHSc, DTM&H, CCFP, FCFP
Emily Wangui Kamau, MBChB, M.Med, MSc (ID)
Patrick Amoth, MBChB, M.Med
Andrew Mulwa, MBChB, Msc
Elizabeth Abong'o, KRCHN
Leonard Kingwara, BSc, MPH, PhD
Dalton C. Wamalwa, MBChB, M.Med, MPH
James Wagude, MBChB, M.Med
Rose Wafula, MBChB, MPH
Lazarus Momanyi, MBChB, MPH
Joseph Nkuranga, MBChB, MSc (Epi)
Florentius Ndinya, MBChB, M.Med,
Anne-Marie Macharia, MBChB, M.Med
Simon Wahome, BPharm, MPharm
Anthony Kiplagat, DCM, BA, MSc
Caroline Wafula, BPharm, MPharm, MBA
Lisa Abuogi, BA, MD, Res, MSc
Rena Patel, BA, M Phil, MD, MPH

Co-Investigators – Mozambique:

Raquel Matavele Chissumba, BSc, MSc, PhD
Patricia Maria Ramgi, MD

Co-Investigators – Tanzania:

Muhammad Bakari, MD, M.Med, PhD
Jamila Said Didi, MD, MBA, M.Med, MSc (Nephrology)

Co-Investigators – Lesotho:

Niklaus Labhardt, MD, MIH, FMH
Anna Klicpera, MD, DTM, MSc
Tapiwa Tarumbiswa MBBS, MBA

Co-Investigators – London School of Hygiene and Tropical Medicine (LSHTM):

Daniel James Grint, BSc, MSc, PhD
Charles Opondo, BPharm, MSc, PhD

1. Introduction

This Charter is for the study titled "Investigating the optimal management of dolutegravir resistance (Ndovu Study)".

The Charter is intended to be a living document. The Data and Safety Monitoring Board (DSMB) may wish to review it at regular intervals to determine whether any changes in procedure are needed..

2. Responsibilities of the DSMB

The DSMB will monitor the main safety and efficacy outcome measures and the overall conduct of the trial, with the aim of protecting the safety and interests of the trial participants.

The primary responsibilities of the DSMB are to: 1) periodically review and evaluate the accumulated study data for participant safety, study conduct and progress, and efficacy, and; 2) make recommendations concerning the continuation, modification, or termination of the study. The members of the DSMB will serve in an individual capacity and provide their expertise and recommendations.

3. Membership

The DSMB will consist of 5 members, each of whom brings one or more of the following qualifications: biostatistician with experience in clinical trials; physician with HIV therapeutics expertise; HIV pharmacist or clinical pharmacologist; investigator with expertise in clinical trials conduct and methodology.

DMSB members will not participate in the study as investigators, nor will be composed of people with no conflicts of interest regarding the study, institutions conducting the trial, study sponsor, the study drug being tested, or any other activity or entity that might affect their objectivity.

Ad-hoc specialists may be invited to participate as non-voting members at any time if additional expertise is desired, as determined by the DSMB Chair.

The DSMB members are:

Walter Jaoko (Chair), MBChB, MTM, MGB, PhD, is a Professor in the Department of Medical Microbiology and Immunology and Director of KAVI-ICR. He has extensive experience in HIV research and has post-graduate training in research ethics. He has led several clinical trials and participated in other DSMB committees.

Andrew Hill, BSc, PhD, is a Senior Visiting Research Fellow at Liverpool University. He also an adviser to the World Health Organization on HIV treatment. He has been the Study Statistician in several large HIV clinical trials and engaged as a DSMB member in several trials.

Gary Maartens, MBChB, FCP, M.Med, is a specialist physician and emeritus professor in the Division of Clinical Pharmacology, University of Cape Town. He has vast experience in TB and HIV research and care and has engaged as technical consultant and expert review committee member at the WHO in this field.

Rajesh Gandhi, BS, MD, is a Professor of Medicine at the Harvard Medical School and the Director of HIV Clinical Services and Education at Massachusetts General Hospital (MGH). He has vast experience in HIV care and research and is site leader of the MGH AIDS Clinical Research Site in the AIDS Clinical Trials Group and Co-Director of the Harvard University Center for AIDS Research.

Eligius Lyamuya, MD, M.Med, PhD, FTAAS, FCPath, FAIS is a Professor of Microbiology and Immunology at the Muhimbili University of Health and Allied Sciences (MUHAS) where he has served in various administrative and academic positions since 1992. He is an active researcher with interests largely in the area of HIV and other sexually transmitted infections and promotion of research ethics. He has served as a PI in several research projects and has over 150 scientific publications in peer reviewed journals.

4. Tasks and Expected Time Commitment

The RCT is expected to run for about 18 months, with a 6-month recruitment period and 12-month of follow-up per participant.

Before the start of study, DSMB members will review the protocol for any major concerns (approximately 2 hours).

During the conduct of the study, the DSMB members will participate in three pre-planned teleconference meetings plus ad-hoc meetings as needed, and develop recommendations during each meeting (each meeting will take approximately 1.5 hours). The meetings will be scheduled to roughly coincide with the following study time points:

- Month 3 (recruitment phase, with some participants reaching month 1 or month 3)
- Month 8 or when two-thirds of participants expected to have completed month 3 visit, using pre-specified interim analysis results
- Month 15 or when all participants have completed their month 6 visit, using the pre-specified primary analysis results
- Ad-hoc Reviews (ad-hoc meetings may be requested by any DSMB member, the study sponsor, the ERCs, or the Chief Investigator at any time to discuss safety concerns. The decision to hold an ad-hoc meeting will be made by the DSMB Chair)

Prior to each meeting (and as required for serious adverse event reports), DSMB members will review summary reports prepared by the Study Statistician and Trial Manager (approximately 1 hour, within one week before each meeting).

5. Meeting Procedures

DSMB meetings shall be held virtually or in-person only if absolutely necessary. Each meeting will require a quorum of 4 DSMB members. The meetings will consist of an Open Session and a Closed Session:

- Open Session
    - Agenda: review of aggregate data on recruitment and follow-up progress; adverse events; site performance and protocol compliance; factors external to the study such as scientific or therapeutic developments that may impact participant safety or the ethics of the study
    - Required Participants: DSMB members, Chief Investigator, Study Statistician
    - Elective Participants: Country Principal Investigators, Co-investigators, Trial Manager or Study Coordinator, Sponsor Representative, ERC or Regulatory Body Representatives, Independent Study Monitor

- Closed Session
    - Agenda: review of safety data; review of efficacy data after the pre-specified interim analysis; recommendations for continuing, modifying or terminating the study
    - Required Participants: DSMB members; the Study Statistician will be available for consultation as needed during the Closed Session
    - Elective Participants: none

The DSMB is responsible for maintaining the confidentiality of its internal discussions and activities.

Each final recommendation should be recorded with the vote tally for the recommendation (how many for, how many against, how many abstain).

Decisions of the DSMB shall be made by consensus. If the meetings fail to reach consents, the issue will be taken to a vote. In that case, each member shall be entitled to one vote per issue. All DSMB members must vote if a given issue requires a vote. If a decision cannot be reached through a vote, the DSMB Chairperson will have the casting vote.

6. Reports Provided to DSMB

The Chief Investigator is responsible for ensuring the DSMB receives the following reports:

- Open Session Reports
    - Aggregate data on recruitment and follow-up progress, adverse events, site performance and protocol compliance: at least one week before each meeting and as requested by the DSMB Chair
    - Data on efficacy: after the pre-specified analyses
    - External study monitor reports: after each study monitor visit
    - Serious Adverse Events: updated cumulative summary
    - Protocol revisions: whenever approved by the ERCs and regulatory bodies

    - Safety reports for the study drug issued by the sponsor, manufacturer or regulatory body
- Closed Session Reports (confidential, password protected, and marked as such)
    - Data on adverse events: at least one week before each meeting and as requested by the DSMB Chair
    - Data on efficacy: after the pre-specified analyses

The DSMB is responsible for maintaining the confidentiality of reports provided to it.

7. Reports from the DSMB

The DSMB Chair is responsible for Summary Reports and Closed Session Reports:

- Summary Report
    - Includes the minutes or brief report from the Open Session and the DSMB's recommendations as to whether the study should continue without change, be modified, or be terminated. Recommendations regarding modification of the design and conduct of the study could include:
        - Changes to informed consent procedures if new risks are identified or suspected risks are refuted
        - Changes to study methods (such as inclusion/exclusion criteria and screening methods)
        - Suspension or early termination of the study because of serious concerns about participants' safety, inadequate performance, or rate of enrollment
        - Suspension or early termination of the study because study objectives have been obtained according to pre-established statistical guidelines
        - Optional approaches for investigators and sponsor to consider when the DSMB determines that the incidence of primary study outcomes is substantially different than expected (such as recommendations to increase the number of study sites or extend the recruitment period)
        - Corrective actions regarding a study site whose performance appears unsatisfactory or appears to raise questions regarding the conduct of the study
    - The summary report will be submitted to the Chief Investigator and sponsor within one month of the meeting. The Chief Investigator and Country Principal Investigators are responsible for submitting all DSMB reports to the respective ERCs and regulatory bodies

- Closed Session Report
    - A confidential summary of the closed session discussions, to be held in the custody of the DSMB Chair until such time as the study is closed, or the DSMB recommends early termination, or the minutes are requested by the regulatory bodies

8. Study Stopping Criteria

The DSMB may recommend terminating either part of, or the entire study for safety or administrative reasons. Potential reasons for stopping include:

- Established efficacy as demonstrated by the protocol defined interim analysis, that is once 241 participants (two-thirds of the study population) have data available on viral suppression at 3 months
- Clear evidence of increased risk of SAEs or deaths
  - The DSMB will review all data related to SAEs and deaths at regular intervals. If the DSMB identifies a discernable pattern of drug-related SAEs or deaths in the study they may recommend the study stop early
- Futility
  - The study may be stopped for continued poor enrolment if it is deemed unlikely that the study will achieve a sufficient sample size to answer the primary hypotheses. Unless the study team provides convincing evidence that changes in recruitment procedures are likely to correct enrolment rates. The study is scheduled to enroll approximately 60 individuals per month. Poor enrolment is defined as enrolment of less than 1/3 of expected participants and will be assessed no sooner than 9 months from study start date
  - In the event of futility, DRT will be conducted on samples stored at baseline to identify baseline DRMs and enrich the pool of those potentially eligible for the RCT

Should the trial sponsor and Chief Investigator agree to terminate part or all of the study, a written statement fully documenting the reasons for such a termination will be provided to the ERCs and regulatory authorities within 15 days.

9. Contact Information of Key Personnel:

Chief investigator: Dr Loice Achieng Ombajo
loisea@uonbi.ac.ke
+254 722 576984

Agreement

I agree to be on the DSMB for the study titled "Investigating the optimal management of dolutegravir resistance (Ndovu Study)" and I am in agreement with the content of this DSMB Charter. I will keep all information gathered during the DSMB meetings strictly confidential.

DSMB members:

Name	Signature	Date
Prof Walter Jaoko (Chair)		
Dr Andrew Hill		
Prof Gary Maartens		
Prof Rajesh Gandhi		
Prof Eligius Lyamuya		
